# Crk Adaptors Negatively Regulate Actin Polymerization in Pedestals Formed by Enteropathogenic *Escherichia coli* (EPEC) by Binding to Tir Effector

**DOI:** 10.1371/journal.ppat.1004022

**Published:** 2014-03-27

**Authors:** Elvira Nieto-Pelegrin, Eugenia Meiler, José Manuel Martín-Villa, María Benito-León, Narcisa Martinez-Quiles

**Affiliations:** 1 Department of Microbiology, School of Pharmacy, Complutense University, Madrid, Spain; 2 Division of Immunology, School of Medicine, Complutense University, Madrid, Spain; Collège de France, France

## Abstract

Infections by enteropathogenic *Escherichia coli* (EPEC) cause diarrhea linked to high infant mortality in developing countries. EPEC adheres to epithelial cells and induces the formation of actin pedestals. Actin polymerization is driven fundamentally through signaling mediated by Tir bacterial effector protein, which inserts in the plasma membrane of the infected cell. Tir binds Nck adaptor proteins, which in turn recruit and activate N-WASP, a ubiquitous member of the Wiskott-Aldrich syndrome family of proteins. N-WASP activates the Arp2/3 complex to promote actin polymerization. Other proteins aside from components of the Tir-Nck-N-WASP pathway are recruited to the pedestals but their functions are unknown. Here we investigate the function of two alternatively spliced isoforms of Crk adaptors (CrkI/II) and the paralog protein CrkL during pedestal formation by EPEC. We found that the Crk isoforms act as redundant inhibitors of pedestal formation. The SH2 domain of CrkII and CrkL binds to phosphorylated tyrosine 474 of Tir and competes with Nck to bind Tir, preventing its recruitment to pedestals and thereby inhibiting actin polymerization. EPEC infection induces phosphorylation of the major regulatory tyrosine in CrkII and CrkL, possibly preventing the SH2 domain of these proteins from interacting with Tir. Phosphorylated CrkII and CrkL proteins localize specifically to the plasma membrane in contact with EPEC. Our study uncovers a novel role for Crk adaptors at pedestals, opening a new perspective in how these oncoproteins regulate actin polymerization.

## Author Summary

Infections by enteropathogenic *Escherichia coli* are an important cause of diarrhea linked to high infant mortality. Such bacteria attach to cells and form actin-rich structures called pedestals, which contain many proteins that play unknown functions during pedestal formation. Here we studied two nearly identical forms (isoforms) of Crk adaptor proteins, CrkII and CrkL, during pedestal formation. Eliminating both isoforms from the cell enhanced pedestal formation, while eliminating only one did not, implying that the isoforms are redundant inhibitors of pedestal formation. We also found that Crk proteins bind the bacterial protein Tir, which binds another adaptor, Nck, to promote actin polymerization in pedestals. We propose that Crk adaptor proteins inhibit actin polymerization by competing with Nck binding to Tir. This work opens the door to investigating how Crk adaptor proteins may participate in numerous actin polymerization pathways.

## Introduction

Enteropathogenic *Escherichia coli* (EPEC) causes infant diarrhea worldwide and is a leading cause of death in developing countries. EPEC adheres to intestinal epithelial cells, causing local disappearance of microvilli and altering cell permeability, giving rise to what are classically known as attaching and effacing (A/E) lesions [1]. At A/E lesions, EPEC attaches to host cells and induces the formation of actin-rich structures called pedestals. Although the ultimate function of pedestals is not completely understood, disrupting genes critical for pedestal formation diminishes colonization and subsequent disease in humans [2] and animal models [3]. Pedestals may facilitate EPEC growth and residence inside the intestine by allowing the bacteria to remain attached to the epithelium during peristalsis and host responses to infection [4].

EPEC uses a type III secretion system to deliver effectors into host cells. One such effector is the translocated intimin receptor, Tir, which drives the major pathway responsible for regulating actin polymerization. Upon injection into the cell cytoplasm, Tir inserts in the plasma membrane, exposing a loop on the cell surface, which in turn binds another bacterial protein, the adhesin intimin [5]. This binding is accompanied by the clustering of Tir and by its phosphorylation on Tyr474 within the C-terminal cytoplasmic domain. This regulatory phosphotyrosine recruits the host cell adaptor protein non-catalytic tyrosine kinase Nck, which in turn recruits N-WASP [6]. Recruitment and activation of N-WASP [7] and of other actin-nucleating proteins such as cortactin [8], [9] leads to Arp2/3 complex-mediated actin polymerization.

Pedestals act as a “molecular niche” to recruit not only actin machinery but many other proteins as well. These proteins include those normally localized to focal adhesions, such as vinculin and talin [10], cell cortex proteins such as ezrin [11] and adaptor proteins such as CT10 regulator of kinase (Crk) proteins [12]. Several excellent reviews have recently been written about EPEC signaling [13], [14], [15].

The first member of the Crk adaptor family to be discovered was v-Crk, a chicken tumor viral oncoprotein that increases tyrosine phosphorylation in cells [16]. The cellular counterpart of v-Crk is CrkII, a proto-oncoprotein that contains an N-terminal Src homology 2 (SH2) domain, referred to as SH2, and two Src homology 3 (SH3) domains, termed N-terminal and C-terminal (referred to as nSH3 and cSH3 respectively). The SH2 domain binds phosphotyrosine motifs [17], and the nSH3 domain binds specific proline-rich motives (for recent reviews see [18], [19]). The cSH3 domain, in contrast, does not bind proline-rich motifs and exerts regulatory activity, mainly in CrkII [20]. The Crk gene gives rise to another splice isoform, CrkI, which lacks a cSH3 domain. In addition, a Crk-like gene called *CrkL*, which maps to a different chromosome, gives rise to an adaptor highly homologous to CrkII [21].

Similar to other adaptor proteins, Crk functions primarily to assemble molecular complexes to distribute signals within the cell [22]. The best studied regulatory mechanism of Crk proteins is the phosphorylation at a tyrosine located in a linker between the N- and C-terminal SH3 domains (Tyr221 in human CrkII, Tyr207 in human CrkL). This tyrosine phosphorylation, carried out mainly by Abl kinase [23], [24], results in an intramolecular interaction between the phosphotyrosine and the SH2 domain of the protein [25]. This interaction favors a conformation that buries the nSH3 domain in CrkII but not in CrkL [26].

In addition to participating in traditionally well-studied processes like cell migration and adhesion [27], Crk adaptors have recently been found to promote apoptosis during endoplasmic reticulum stress [28] and to regulate the immunological synapse [29]. Crk adaptors have been implicated in many types of human cancer [30] and in embryonic development in studies involving CrkI/II [31] and CrkL known-down mice [32]. The latter mice are a model of human DiGeorge syndrome, in which congenital heart disease and immune deficiency are frequently present.

CrkII contributes to bacterial invasion of epithelial cells by *Shigella* [33] and *Salmonella* [34], and it is a target of virulence factors. These findings, together with the report that CrkII localizes to EPEC pedestals [12], led us to hypothesize that it may play a role in EPEC infection. Here we investigated the function of Crk adaptors during EPEC pedestal formation.

## Results

### Effect of siRNA-induced inhibition of CrkI/II and CrkL expression on EPEC pedestal formation

**Figure 1:**
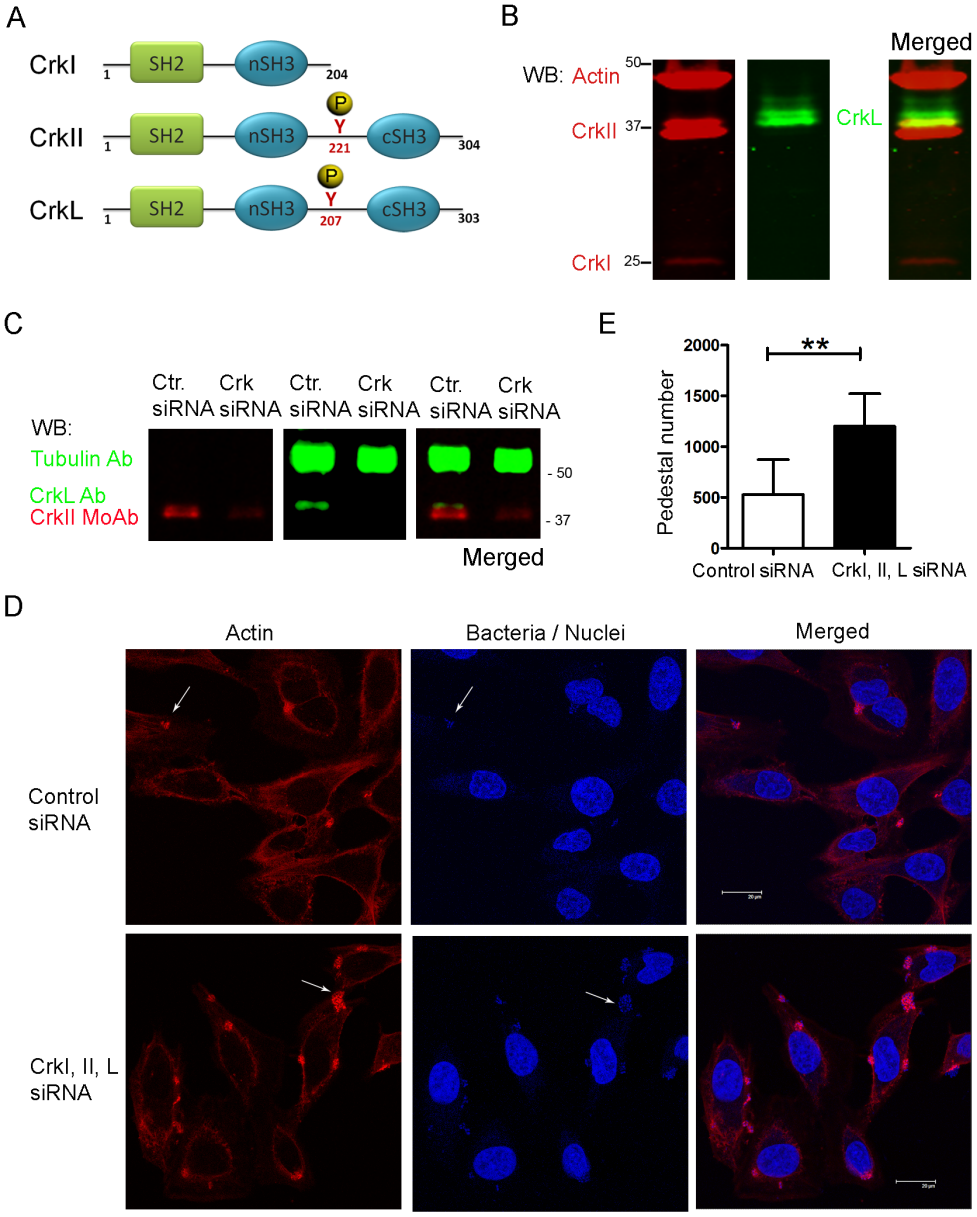
Pedestal formation in HeLa cells treated with siRNA to reduce CrkI/II and CrkL expression. (**A**) Schematic representation of Crk proteins under investigation. (**B**) WB of HeLa cell extracts using anti-CrkI/II MoAb and anti-CrkL Ab and the Odyssey imaging system to show expression levels of the corresponding proteins, or (**C**) to show decreased Crk expression in infected cells pretreated with siRNA. As a loading control the blots were probed with anti-actin monoclonal Ab (MoAb) or anti-tubulin Ab (upper bands). (**D**) Confocal fluorescence images of HeLa cells pretreated with siRNA against CrkI/II and CrkL or control siRNA and infected with preactivated EPEC for 2 h at an MOI of 3. Actin was stained with TRITC-phalloidin (red), while bacteria were stained with DAPI (blue). Arrows point at pedestals and bacteria. The scale bar represents 20 μm. (**E**) Quantitation of the number of pedestals on infected HeLa cells pretreated using siRNA with two oligonucleotides against CrkI/II and CrkL (black bar) compared to control oligonucleotide treated cells (white bar). Quantitation was done by counting the number of pedestals on 100 cells. Data in the graph show mean ± standard deviation (SD) for three independent experiments. The difference between groups was statistically significant based on Students *t*-test analysis; **, p<0.01.

Crk is an important adaptor molecule that participates in diverse signaling pathways and that localizes to EPEC pedestals [12]. To assess the role of Crk adaptors during EPEC pedestal formation, we knocked-down the expression of all Crk isoforms in HeLa cells using commercially available oligonucleotides specific for CrkI/II and L (Fig. 1). In Fig. 1A we show a schematic representation of the Crk adaptor family. In HeLa cells, endogenous CrkII and CrkL were readily detected, whereas CrkI was expressed at nearly undetectable levels (Fig. 1B), consistent with previous work [35]. At 20 h after siRNA transfection, CrkII and CrkL levels were reduced by 73% and 67% (average of three experiments after normalizing for tubulin), as quantified by western blotting (WB, Fig. 1C). At that time point, we infected the cells with EPEC and allowed pedestal formation. Fig. 1D shows immunofluorescence staining of polymerized actin with TRITC-phalloidin and of bacteria with DAPI. The number of pedestals was significantly higher in cells treated with anti-Crk siRNAs than in cells treated with control siRNA in three different experiments (Fig. 1E). This unexpected result indicates that simultaneous down-regulation of CrkI/II and CrkL expression potentiates pedestal formation by EPEC. However, down-regulation of either CrkII or CrkL on its own did not significantly affect pedestal numbers (Fig. S1), suggesting that Crk isoforms act redundantly to inhibit pedestal formation. We cannot exclude the possibility that this negative result is due to the fact that our system inhibited CrkII or CrkL expression by only 76% and 53%, respectively.

### Effect of expression of Crk mutants on pedestal formation

**Figure 2:**
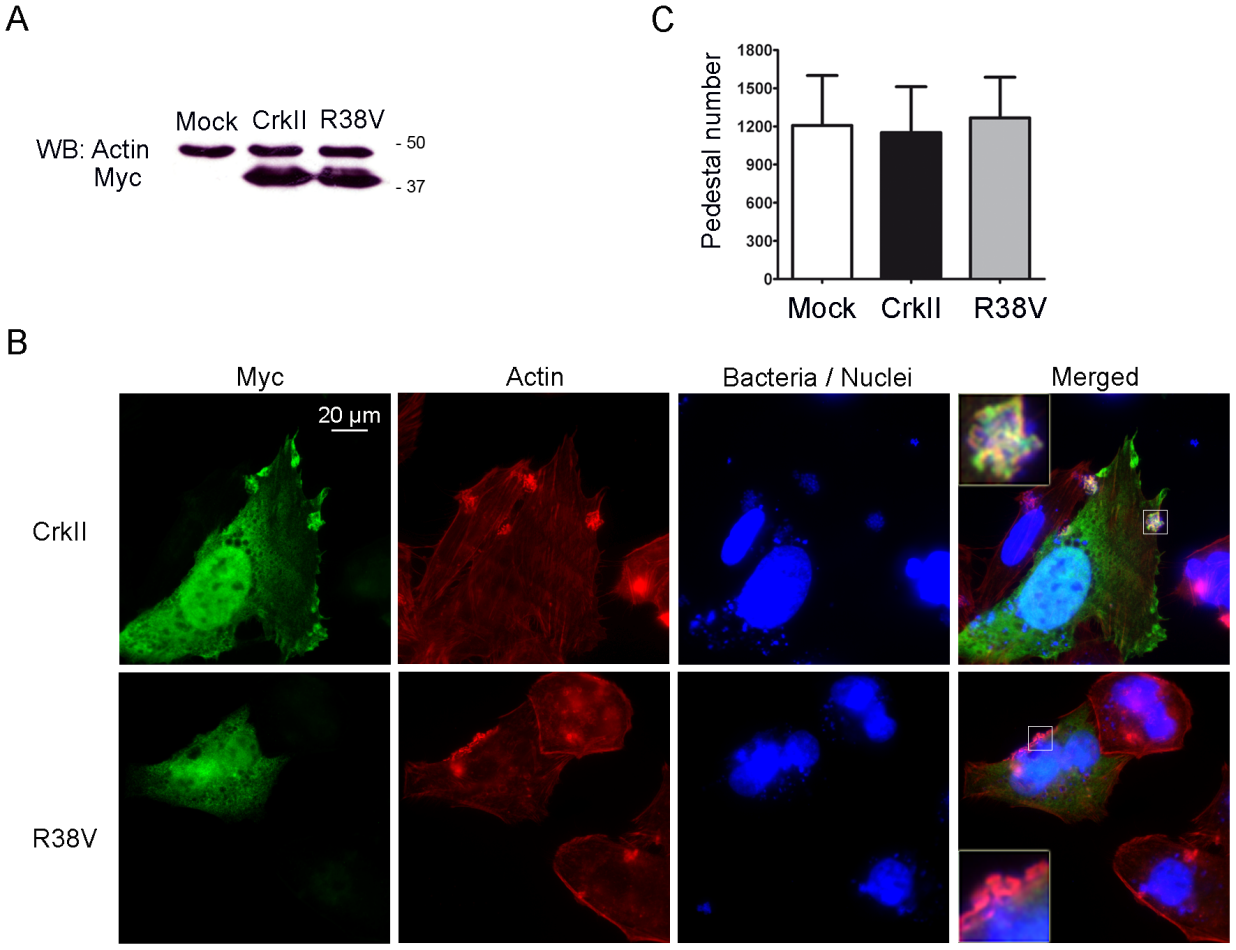
Pedestal formation in HeLa cells expressing a dominant-negative CrkII mutant. (**A**) Expression of Crk in transfectants was assessed by WB with anti-Myc MoAb. Blots were probed with anti-actin Ab as a loading control. (**B**) Immunofluorescence images of HeLa cells transfected with Wild-Type (WT) CrkII, a CrkII dominant-negative mutant (R38V), or transfection reagent alone (mock; data not shown), then infected with preactivated EPEC for 2 h at an MOI of 3. Myc-tagged Crk constructs were visualized in green using anti-Myc MoAb, followed by anti-mouse Alexa-488 secondary Ab. Actin was stained in red using TRITC-phalloidin, while bacteria were stained blue using DAPI. The merged images shown were generated using AxioVision software. Insets are 4× digital zoom images. (**C**) Quantitation of the number of pedestals on mock-treated HeLa cells (white bar), cells overexpressing CrkII (CrkII, black bar) or cells overexpressing the dominant-negative R38V CrkII mutant (R38V, grey bar). Quantitation was done by counting the number of pedestals on 100 cells. The graph shows mean ± standard deviation (SD) for three independent experiments. The differences among the groups were not statistically significant based on Student's *t*-test analysis.

To gain further insights in the way Crk adaptors participate in pedestal formation, we performed experiments in HeLa cells transfected with a Myc-tagged dominant-negative CrkII mutant carrying an R38V mutation in the SH2 domain (Fig. 2). This Arg is invariant in SH2 domains and is essential for recognizing phosphotyrosine. Mutating it abolishes Crk interaction with binding partners [36]. As a control, we used wild-type (WT) Myc-CrkII. Fig. 2A shows that WT CrkII and R38V mutant were expressed in transfected cells at similar levels, as assessed by WB using an anti-Myc monoclonal antibody (MoAb). Fig. 2B shows immunofluorescence staining of HeLa cells transfected with WT or R38V CrkII constructs and infected with EPEC. As a negative control, cells were treated with transfection reagent only (data not shown). We used anti-Myc MoAb followed by anti-mouse Alexa 488 secondary antibody (Ab) to visualize Myc-tagged Crk constructs in green, TRITC-phalloidin to label pedestals in red, and DAPI to label EPEC in blue. In agreement with the previously described localization of endogenous CrkII protein [12], the transfected Myc-tagged WT CrkII localized to pedestals, while the R38V CrkII mutant did not. Fig. 2C shows that the number of pedestals did not differ among the groups in three different experiments. These results indicate that neither overexpression of WT CrkII nor inhibition of endogenous CrkII using a dominant-negative mutant significantly affects pedestal formation. The results also indicate that localization of Crk proteins to pedestals is mediated mainly by their SH2 domain.

**Figure 3:**
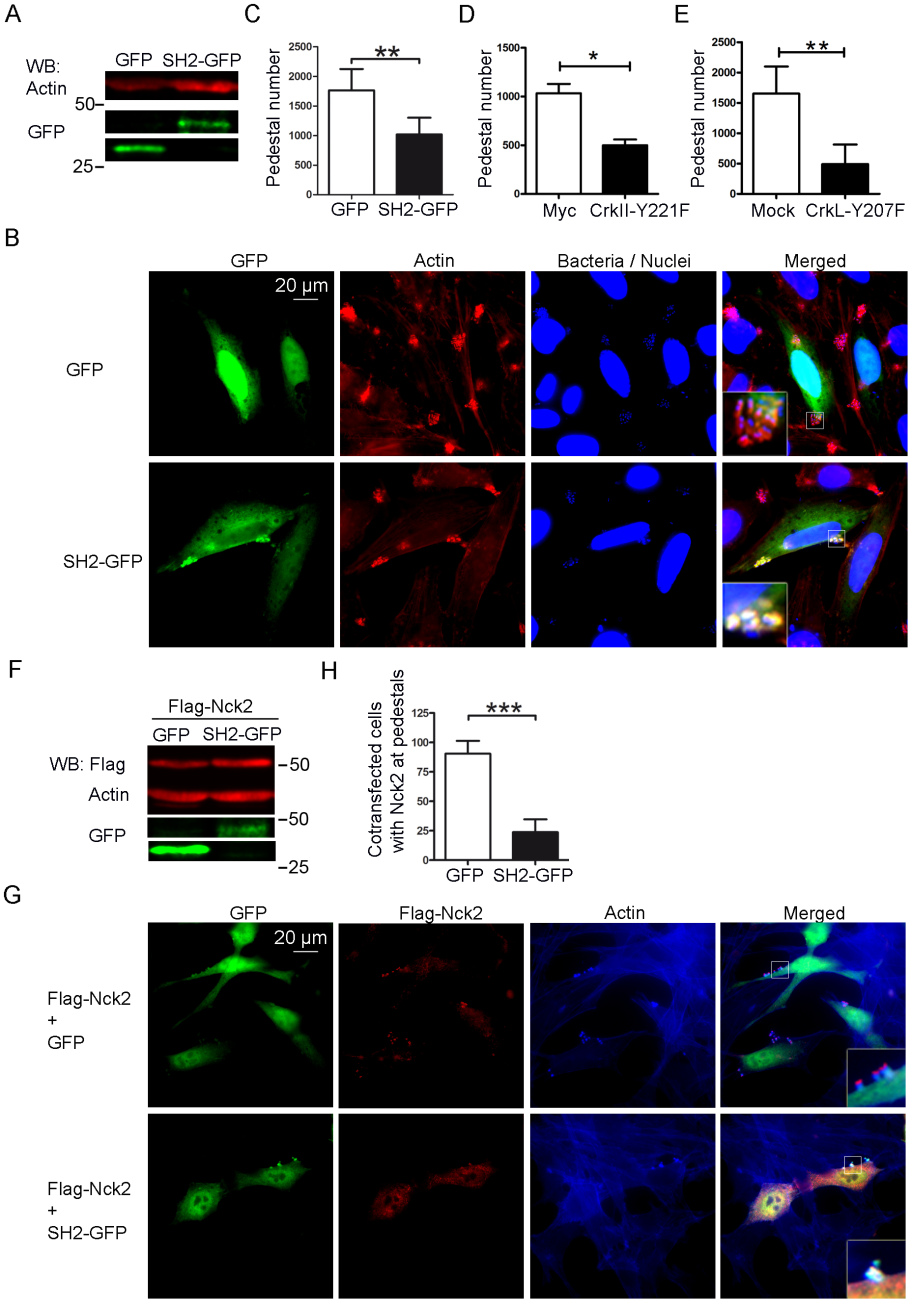
Pedestal formation in cells expressing Crk mutants. (**A**) Expression of GFP alone (GFP) or GFP-tagged CrkII SH2 domain (SH2-GFP) in transfectants was assessed by WB with anti-GFP Ab. Blots were probed with anti-actin MoAb as a loading control. (**B**) Fluorescence images of HeLa cells transfected with GFP or SH2-GFP and then infected with preactivated EPEC for 2 h at an MOI of 3. GFP-tagged constructs appear green; actin, red (TRITC-phalloidin) and EPEC, blue (DAPI). The merged images shown were generated using AxioVision software. Insets are 4× digital zoom images. (**C**) Quantitation of the number of pedestals on cells expressing GFP or SH-GFP. Quantitation was done by counting the number of pedestals on cells expressing the constructs in six experiments. (**D, E**) HeLa cells were transfected with Myc-tagged empty vector or with plasmids encoding Myc-CrkII-Y221F or CrkL-Y207F phosphorylation-deficient mutants, and then infected with preactivated EPEC at an MOI of 3. Pedestal number was compared in cells expressing Myc and Myc-CrkII-Y221F or between Mock-transfected cells and CrkL-Y207F transfected cells in three different experiments. (**F**) WB of Nck1/2-deficient MEFs transfected with Flag-Nck2 plasmid as well as a plasmid encoding either GFP alone or GFP-tagged CrkII SH2. (**G**) Immunofluorescence staining of transfected Nck1/2-deficient MEFs after infection with EPEC at an MOI of 225. Cells were stained with anti-Flag MoAb followed by Alexa 568-conjugated goat anti-mouse Ab. F-actin was visualized with Alexa 350 Phalloidin. GFP expression is shown in green. (**H**) The number of cells in three different experiments showing Flag staining at pedestals was counted among cells expressing empty GFP or GFP-tagged CrkII SH2 and normalized to 100. The graph shows mean ± standard deviation (SD) for three independent experiments. The differences among the groups were statistically significant based on Student's *t*-test; *, p<0.05, **, p<0.01, ***, p<0.001.

Expression of the isolated SH2 domain of Nck1/2 inhibits pedestal formation [6]. Therefore we investigated whether we could also inhibit pedestal formation by expressing the isolated SH2 domain of CrkII. We transfected cells with a plasmid encoding the SH2 domain of CrkII fused to GFP (referred as to SH2-GFP) or with empty GFP vector (GFP) as a negative control, and we quantified the number of pedestals (Fig. 3). Fig. 3A shows that both constructs were expressed in transfected cells, as assessed by WB using an anti-GFP polyclonal Ab. Fig. 3B shows fluorescence staining of HeLa cells transfected with GFP or SH2-GFP constructs and infected with EPEC. GFP expression is shown in green, TRITC-phalloidin staining of polymerized actin in red and DAPI staining of bacteria and nuclei in blue. Fig. 3C shows that the number of pedestals was significantly lower in cells expressing the SH2-GFP fusion protein. Non-transfected cells showed similar numbers of pedestals as cells expressing GFP alone (data not shown). These results indicate that overexpression of the isolated CrkII SH2 domain inhibits pedestal formation and further suggests that the localization of Crk proteins to pedestals is probably mediated by their SH2 domain.

To probe further whether the SH2 domain of CrkII and CrkL inhibit pedestal formation, we took advantage of the fact that the binding ability of this domain depends on phosphorylation of tyrosines 221 and 207, respectively. This tyrosine phosphorylation allows the intramolecular interaction between the phosphotyrosine and the SH2 domain. HeLa cells were transfected with constructs expressing phosphorylation deficient Myc-tagged CrkII-Y221F or untagged CrkL-Y207F constructs and infected with EPEC (Fig. 3D, E). Control cells were transfected with empty Myc vector or were treated only with the transfection reagent (mock conditions). Expression of the constructs was verified by WB and the number of pedestals obtained with each construct was quantified by immunofluorescence (data not shown). We found that both mutants inhibited pedestal formation significantly (Fig. 3D, E). Together these results indicate that the SH2 domain mediates the ability of Crk adaptors to inhibit pedestal formation, although we cannot exclude that other adaptor domains also contribute.

Given that the SH2 domain of CrkII inhibits pedestal formation, we wondered whether it interferes with Nck recruitment to pedestals (Fig. 3F–H). We tested this hypothesis using Nck1/2-deficient mouse embryonic fibroblasts (MEFs) because these cells allow pedestal formation mainly when reconstituted with Nck1 or Nck2 [6], because there is a secondary Tir-dependent and Nck-independent pathway to actin [37]. Therefore we co-transfected Nck1/2-deficient MEFs with both Flag-tagged Full-Length Nck2 and either GFP alone (Flag-Nck2 + GFP) or SH2-GFP CrkII (Flag-Nck2 + SH2-GFP); expression of all constructs was confirmed by WB (Fig. 3F). Immunofluorescence staining was performed using anti-Flag MoAb, followed by Alexa 568-conjugated anti-mouse goat Ab (in red) and Alexa 350 Phalloidin (in blue); GFP was visualized in green (Fig. 3G). We determined the number of co-transfected cells that were positive for Flag staining at pedestals; this number was significantly lower in cells expressing both Flag-Nck2 and SH2-GFP than in cells expressing Nck2 and GFP alone (Fig. 3H). In addition, Nck2 staining in these cells was concentrated in the cell cytoplasm and relatively delocalized from pedestals, whereas it was highly localized to pedestals in control cells (Fig. 3, S5). Together these results suggest that Crk isoforms may inhibit pedestal formation by blocking Nck recruitment to pedestals.

### Analysis of Crk adaptor contribution to pedestal formation using CrkI/II and CrkL deficient cells

To test whether CrkII and CrkL adaptors can compensate for each other in the siRNA and the R38V dominant-negative experiments in Figs. S1 and 2, we performed EPEC infection experiments using CrkI/II and CrkL-deficient MEFs (CrkI/II -/-, CrkL -/-; Fig. S2). In addition, we tried to distinguish between CrkI and CrkII contributions by transfecting CrkI/II -/- MEFs with WT Myc-CrkII to reconstitute CrkII expression; the resulting cells were referred to as Rescued CrkII (R CrkII). The number of pedestals did not significantly differ among WT, CrkI/II -/-, CrkL -/- or Rescued MEFs (Fig. S2C, E), confirming the results obtained with HeLa cells (Figs. S1).

**Figure 4:**
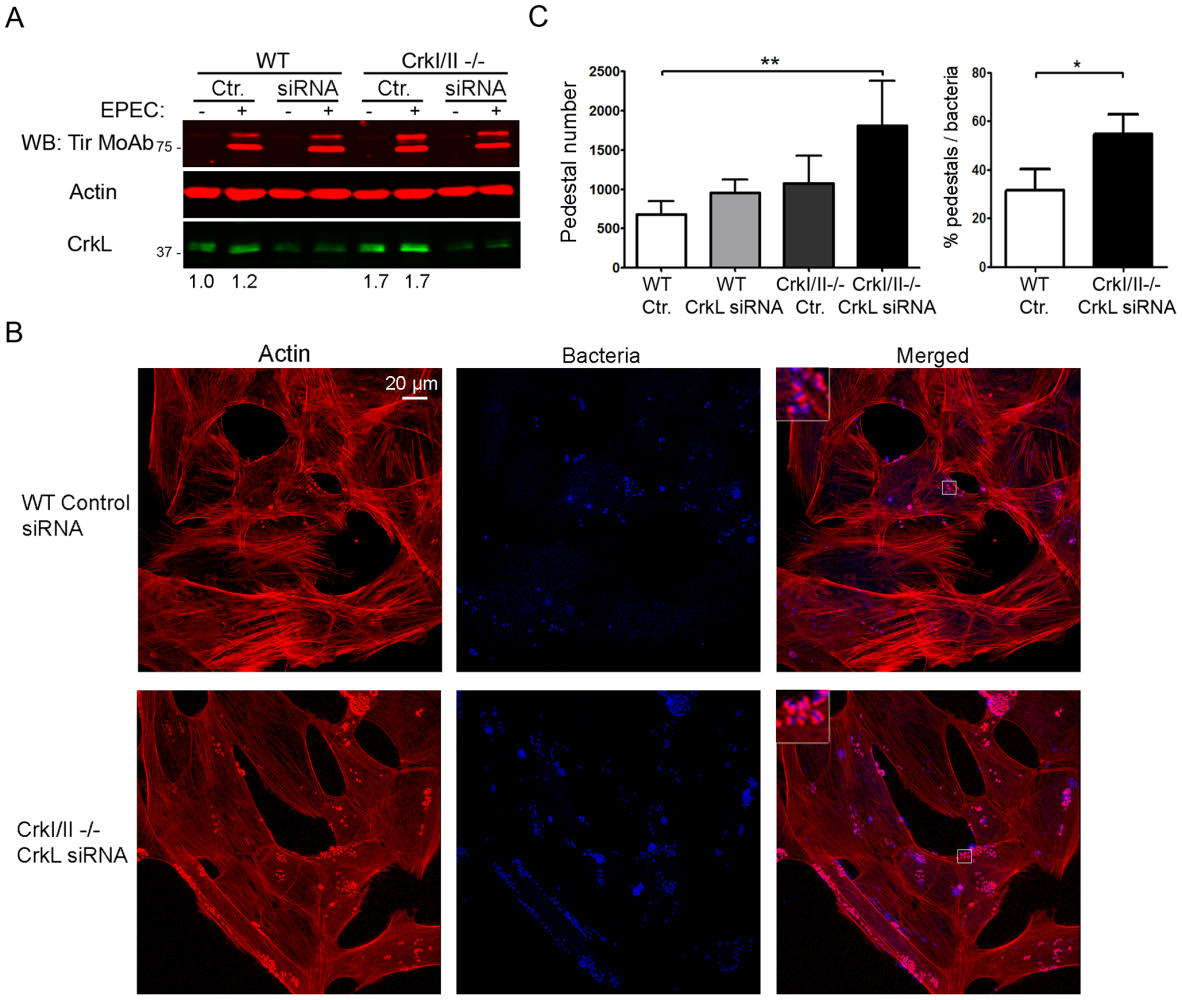
Down-regulation of CrkL expression by siRNA in CrkI/II-deficient MEFs potentiates pedestal formation. (**A**) WB with anti-CrkL Ab in WT and CrkI/II-deficient MEFs to show lower CrkL levels in cells treated using siRNA with an oligonucleotide against CrkL than in cells treated with a control oligonucleotide (lower bands). The levels of injected Tir effector were assessed by WB with anti-Tir MoAb. As a loading control, blots were probed with anti-actin MoAb. (**B**) Immunofluorescence images of WT and CrkI/II-deficient MEFs in which CrkL expression was inhibited by siRNA; cells were infected with preactivated EPEC for 3 h at an MOI of 3. Actin was stained red with TRITC-phalloidin, while EPEC was stained blue using anti-LPS Ab followed by Alexa 405-conjugated goat anti-mouse secondary Ab. Confocal images were merged using Leica software. Insets are 4× digital zoom images. (**C**) Quantitation of the number of pedestals and the ratio of pedestals to bacteria on WT and CrkI/II-deficient cells treated using siRNA with a scrambled control (Ctr.) oligonucleotide (white and dark-grey bars, respectively) or with an oligonucleotide to reduce CrkL expression (grey and black bars, respectively). The number of pedestals was quantified based on counts with 100 cells. The ratio of pedestals to bacteria was quantified by counting the number of adhered bacteria with or without pedestals on 50 cells; the resulting ratio was expressed as a percentage. The graph shows mean ± standard deviation (SD) for three independent experiments. The indicated groups differed significantly based on Student's *t*-test. *, p<0.05; **, p<0.01.

While the results of the experiments in Figs. S1 and S2 are consistent with the idea that CrkI/II and CrkL are involved in pedestal formation, they do not provide direct evidence of such involvement, since CrkL may be compensating for down-regulation or loss of CrkI/II and *vice versa*. To test for the involvement of CrkI/II and CrkL more directly, we used siRNA to inhibit the expression of CrkL in CrkI/II-deficient MEFs and then we infected the cells with EPEC (Fig. 4). The expression levels of CrkL were reduced by 60%, as quantified by WB. To control for possible differences in infection, we confirmed that the levels of Tir translocated by EPEC were similar under the different treatment conditions (Fig. 4A, data not shown). We stained the cells for actin using TRITC-phalloidin. To stain for EPEC, we chose not to use DAPI in order to avoid overexposure of cell nuclei; instead, we used a mouse anti-*E. coli* lipopolysaccharide (LPS) MoAb followed by an Alexa 405-conjugated anti-mouse secondary Ab (blue; Fig. 4B). We found that cells expressing none of the three Crk isoforms formed significantly more pedestals than did WT cells (Fig. 4C). These results were confirmed using a second siRNA oligonucleotide to inhibit CrkL expression in CrkI/II-deficient MEFs (Fig. S3A–C).

To evaluate whether the observed increase in pedestal number reflected a direct effect on actin polymerization and not to an improvement in bacterial adhesion, we quantified the fraction of bacteria that generated pedestals in CrkI/II-deficient MEFs depleted in CrkL by siRNA (Fig. 4C). We found that cells depleted of CrkI, CrkII and CrkL showed a higher ratio of pedestals to bacteria than did their WT counterparts, indicating more efficient pedestal formation. This supports the idea that depleting Crk isoforms favors actin polymerization in pedestals.

**Figure 5:**
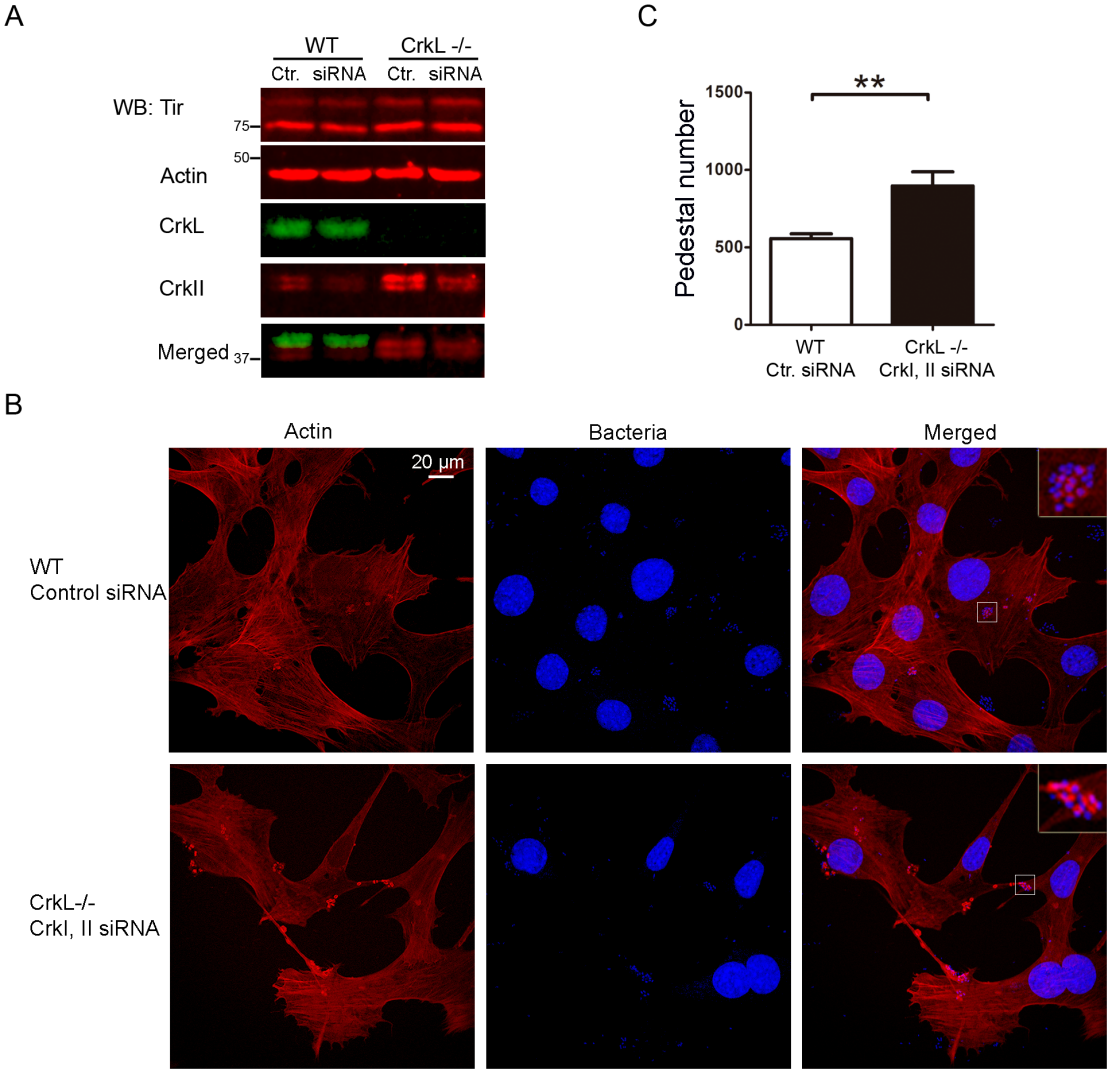
Down-regulation of CrkI/II expression by siRNA in CrkL-deficient MEFs enhances pedestal formation. (**A**) WB with anti-CrkII Ab in WT and CrkL-deficient MEFs to show lower CrkII levels in cells treated using siRNA with an oligonucleotide against CrkI/II than in cells treated with control (Ctr.) oligonucleotide. The blot was probed with anti-CrkL Ab as a control for cell genotype, and with anti-actin MoAb as a loading control. The levels of Tir effector are shown. (**B**) Fluorescence images of WT and CrkL-deficient MEFs in which CrkI/II expression was inhibited by siRNA; cells were infected with preactivated EPEC at an MOI of 3. Actin was stained red with TRITC-phalloidin, while EPEC was stained blue using DAPI. Images were merged using Leica software. Insets are 4× digital zoom images. (**C**) Quantitation of the number of pedestals on WT and CrkL-deficient cells treated for siRNA using a scrambled control (Ctr.) oligonucleotide (white bar) or an oligonucleotide to reduce CrkI/II expression (black bar). Quantitation was done by counting the number of pedestals on 100 cells. The graph shows mean ± standard deviation (SD) for three independent experiments. The indicated groups differed significantly based on Student's *t*-test. **, p<0.01.

To further confirm the results obtained in CrkI/II-deficient cells in which CrkL was depleted by siRNA with two oligonucleotides (Figs. 4 and S3A–C), we expressed the dominant negative R38V CrkII mutant in CrkI/II-deficient MEFs (Fig. S3D). These cells formed a significantly greater number of pedestals than did WT and CrkI/II-deficient MEFs transfected with Myc alone (Fig. S3E). Next we used siRNA to inhibit the expression of CrkI/II in CrkL-deficient MEFs after which the cells were infected with EPEC (Fig. 5). The expression levels of CrkI/II were reduced by 50%. As a control for possible differences in infection, we confirmed that the levels of injected Tir were similar under the different treatment conditions (Fig. 5A). We stained the cells to determine the number of pedestal formed after infections. Although the siRNA treatment reduced CrkI/II expression only by 50%, CrkL-deficient MEFs treated by siRNA formed significantly more pedestals than did WT cells (Fig. 5B, C). We confirmed these results using a second siRNA oligonucleotide to inhibit CrkI/II expression in MEFs deficient in CrkL (data not shown). Taken together, the results of these siRNA experiments in MEFs deficient in CrkI/II and CrkL suggest that Crk isoforms inhibit pedestal formation redundantly, with one isoform able to compensate for the absence of others.

In light of this redundant inhibition of pedestal formation, we wondered whether MEFs deficient in CrkI/II or CrkL might constitutively upregulate the remaining Crk isoform. To address this question, we prepared lysates from MEFs deficient in either CrkI/II or CrkL, and we blotted them with anti-CrkI/II and CrkL Abs simultaneously using the Odyssey Scan system (Fig. S4A, C). To our surprise, MEFs deficient in CrkI/II or CrkL expressed significantly higher basal levels of the remaining isoform (Fig. S4B, D). To our knowledge, this is the first such analysis of CrkI/II or CrkL-deficient cells, and the findings may explain why the numbers of pedestals formed by EPEC in cells expressing at least one Crk isoform did not significantly vary in our experiments (Figs. 2, 4, and Figs. S1 and S2).

### EPEC pedestal formation induces Crk adaptor phosphorylation

**Figure 6:**
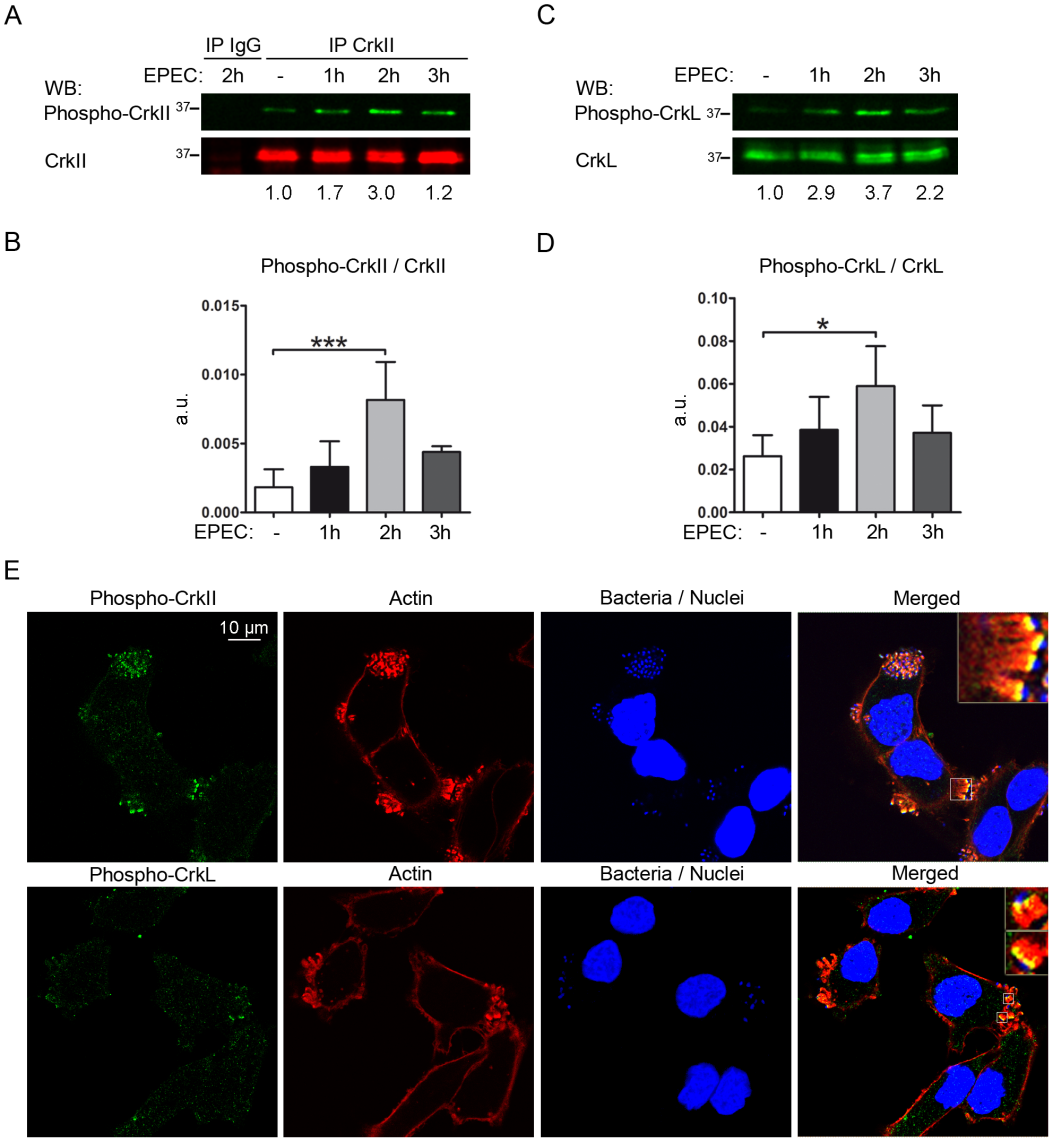
EPEC infection induces tyrosine phosphorylation of Crk adaptor proteins. HeLa cells were serum-starved for 16 h prior to infection with preactivated EPEC for 1, 2 or 3 h at an MOI of 45. The basal level of phosphorylation of Crk proteins was visualized in uninfected cells (- EPEC). (**A**) CrkII or IgG isotype control immunoprecipitates were probed by WB with a phosphospecific Ab against phospho-Tyr221 in CrkII (phospho-CrkII) to show induction of phosphorylation. The blot was also probed with anti-CrkI/II MoAb to show total levels of CrkII. The ratio of phospho-CrkII to non-phosphorylated CrkII in a representative experiment is shown. (**B**) Statistical analysis of the ratio of phospho-CrkII to CrkII signals with respect to the basal level in uninfected cells using one-way ANOVA with Dunnett test. The graph shows mean ± SD for four independent experiments. a.u.: arbitrary units. ***, p<0.001. (**C**) WBs with a phosphospecific Ab against phospho-Tyr207 in CrkL to show the induction of phosphorylation, and with anti-CrkL Ab to show total levels of CrkL. The ratio of the phospho-CrkL to CrkL in a representative experiment is shown. (**D**) Statistical analysis of the ratio of phospho-CrkL to CrkL levels with respect to the basal level at 0 h using one-way ANOVA with Dunnett test. The graph shows mean ± SD for four independent experiments. a.u.: arbitrary units. *, p<0.05. (**E**) Immunofluorescence images of HeLa cells that were starved for 16 h prior to infection with preactivated EPEC for 3 h at an MOI of 15. Immunofluorescence staining was done using phosphospecific Abs against Tyr221 in CrkII (phospho-CrkII) or Tyr207 in CrkL (phospho-CrkL) followed by Alexa 488-conjugated goat anti-rabbit secondary Ab (green). Actin was stained red using TRITC-phalloidin; bacteria were stained blue using DAPI. Pictures were taken on a confocal microscope and images from one section are shown, together with 4× digital zoom images (insets). Images were merged using Leica software.

EPEC attachment to cells promotes the redundant activation of tyrosine kinases, including Src family kinase c-Fyn [38] and Abl/Arg [39]. Since Abl is the major kinase regulating the Crk adaptor family [23], we wondered whether EPEC attachment to cells induces Crk phosphorylation on the major regulatory tyrosines, which would allow them to interact intramolecularly with the SH2 domain (Fig. 1A). We performed EPEC infections of HeLa cells for 1, 2 and 3 h at two multiplicities of infection (MOIs), and analyzed the phosphorylation status of Tyr221 in CrkII and Tyr207 in CrkL using commercially available phosphospecific Abs (Fig. 6 and data not shown). Because the signal was weaker for phospho-CrkII than for phospho-CrkL we performed immunoprecipitations of total CrkII, and blotted them with anti-phophoY221-CrkII Ab. PhosphoY207-CrkL was detected directly by WB. In order to improve our ability to detect signal induction, cells were starved overnight in serum-free medium prior to infection, and the basal level was visualized in uninfected cells (Fig. 6A and C). The phosphospecific signal was quantified by normalizing it to the signal of the corresponding non-phosphorylated protein (Fig. 6B and D). EPEC infection induced a significant increase in CrkII-Tyr221 and CrkL-Tyr207 phosphorylation that peaked at 2 h of infection and decayed thereafter at an MOI of 45 (Fig. 6A–D) while the levels peaked at 3 h at a lower MOI of 3 (data not shown). These results indicate that EPEC induces transient phosphorylation of CrkII and CrkL adaptors at the major regulatory tyrosines 221 and 207, respectively. Maximal phosphorylation correlates with MOI.

### Recruitment of phosphorylated Crk adaptors to pedestals

Although a previous study has localized Crk adaptors to pedestals [12], whether the corresponding phosphorylated adaptors are recruited to EPEC pedestals is unknown. To address this question, we performed confocal immunofluorescence experiments to visualize phospho-Crk adaptors in EPEC pedestals at 3 hours of infection (Fig. 6). For staining, we used the phosphospecific anti-pY221 Ab followed by anti-rabbit Alexa 488-conjugated secondary Ab (green), while actin was visualized with TRITC-phalloidin (red) and bacteria with DAPI (blue; Fig. 6E). Pictures were taken on a confocal microscope and merged using the manufacturer's software. The merge of the three images clearly shows that phospho-CrkII does not localize along the entire length of the pedestal but rather to a thin layer restricted to the region of plasma membrane in contact with the bacteria. We next stained for pTyr207-CrkL as described above and found its localization to be similar to that of phospho-CrkII (Fig. 6E). Similar results were obtained in immunofluorescence experiments performed at 2 hours of infection (data not shown). In addition, we performed immunofluorescence experiments to visualize phospho-Crk proteins and bacteria as a control (data not shown). Taken together, the immunofluorescence results show that phosphorylated CrkII and CrkL localize to pedestals, where they are specifically enriched at thin areas near the plasma membrane in contact with EPEC rather than along the entire pedestal stalk.

### Binding of the SH2 domain of CrkII and CrkL to Tir depends on phosphorylation of Tir tyrosine 474

**Figure 7:**
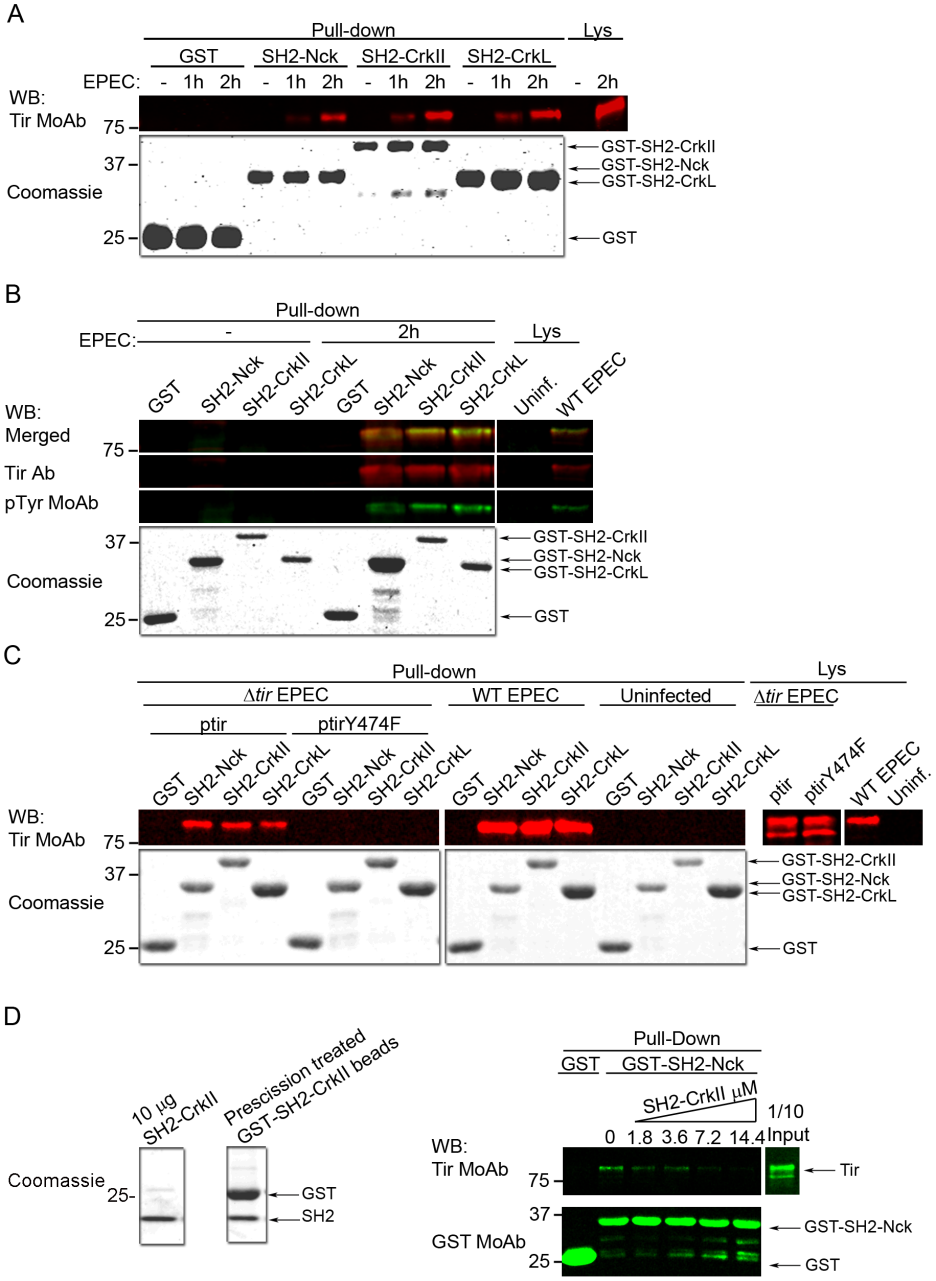
The SH2 domains of CrkII and CrkL compete with Nck to bind Tir at its phosphorylated tyrosine 474. For pull-down experiments, total cell lysates were mixed with the following GST fusion proteins: GST alone, GST-SH2-Nck, GST-SH2-CrkII or GST-SH2-CrkL. As a control, 1:20 of the total amount of cell lysate used for each pull-down assay was analyzed (Lys). The GST fusion proteins used in the pull-down assays were analyzed by Coomassie blue staining; the amounts were 1:20 the amounts used in the pull-down experiments. These experiments were performed at least three times. (**A**) HeLa cells were left uninfected (- EPEC) or infected with preactivated EPEC for 1 or 2 h at an MOI of 180. Pull-down experiments were analyzed by WB using an anti-Tir MoAb. (**B**) HeLa cells were left uninfected (- EPEC) or infected with preactivated EPEC for 2 h at an MOI of 180. Pull-down experiments were analyzed by WB using an anti-Tir polyclonal Ab and a generic anti-phophotyrosine MoAb. (**C**) HeLa cells were left uninfected (- EPEC) or infected for 2 h at an MOI of 150 with preactivated Δ*tir* mutant EPEC complemented with a low-copy-number plasmid expressing WT Tir (p*tir*) or Tir with a Tyr474Phe substitution (p*tir*Y474F). Pull-down experiments were analyzed by WB using an anti-Tir polyclonal Ab. As a control, infections with WT EPEC were performed in parallel. (**D**) Binding of recombinant CrkII SH2 domain to Tir competes with the binding of the SH2 domain of Nck. Recombinant CrkII SH2 domain was excised using PreScission protease from the GST-SH2 domain coupled to GSH beads. The indicated amounts of soluble SH2 domain were added to infected HeLa cell lysates. After incubation, pull-downs were performed by adding empty GST or GST-SH2 Nck, and then visualized by WB with sequential blotting with anti-Tir and anti-GST MoAbs.

The SH2 domain of Nck1/2 binds Tir through its phosphorylated Tyr474 [6], activating the major Tir-Nck-N-WASP pathway, which promotes actin polymerization. Since Crk adaptors also have an SH2 domain, we wondered whether they bind Tir in a similar manner as Nck and whether this might help explain how Crk adaptors inhibit EPEC pedestal formation. We expressed and purified all three SH2 domains as GST fusion proteins, as verified using Coomassie blue staining (Fig. 7). We then performed pull-down assays using the isolated SH2 domains of CrkII and CrkL and lysates of HeLa cells infected by EPEC for different periods. The SH2 domain of Nck was used as a positive control for Tir interaction, and the isolated GST protein as a negative control. Membranes were blotted with an anti-Tir MoAb (Fig. 7A). The results show that the SH2 domain of CrkII and CrkL efficiently pulls down Tir, to a similar degree as the SH2 domain of Nck.

SH2 domains bind proteins in a phosphotyrosine-dependent manner [18]. To confirm that the Tir molecules pulled-down by the SH2 domains of CrkII or CrkL were tyrosine-phosphorylated as expected, we performed pull-down experiments in which we simultaneously detected Tir and its corresponding tyrosine phosphorylation using an anti-Tir polyclonal Ab and a generic anti-phosphotyrosine MoAb respectively. The merge of Tir and phosphotyrosine WB images (Fig. 7B) shows that Tir molecules pulled-down by the SH2 domain of CrkII or CrkL were indeed tyrosine-phosphorylated.

As previously mentioned, the Nck SH2 domain specifically binds Tir through its phosphotyrosine 474 [6]. Therefore we sought to establish whether the SH2 domains of CrkII or CrkL bind Tir at the same phosphotyrosine (Fig. 7C), which would explain the competition we observed. To this end, we infected HeLa cells for 2 h with an EPEC strain that lacks Tir (Δ*tir* EPEC mutant). The Δ*tir* EPEC was complemented with a low-copy-number plasmid that expresses Tir carrying a Tyr474Phe substitution that cannot be phosphorylated (p*tir*Y474F). As controls, we infected cells with the Δ*tir* EPEC mutant complemented with WT Tir plasmid (p*tir*), and we infected cells with WT EPEC or we left cells uninfected. Cell lysates were subjected to pull-down assays using the isolated SH2 domain of CrkII, CrkL or Nck. WB analysis with anti-Tir MoAb revealed that the SH2 domain of CrkII or CrkL did not pull-down the Y474F Tir mutant. In contrast, they efficiently pulled down Tir when HeLa cells were infected with the Δ*tir*+p*tir* combination or with WT EPEC. The findings in Fig. 7A–C suggest that CrkII and CrkL can bind Tir through its phosphorylated Tyr474 during EPEC infection and therefore compete with Nck for binding Tir.

To further characterize the Tir-Crk complexes, we performed competitive pull-down experiments (Fig. 7D). We added increasing amounts of purified recombinant CrkII SH2 domain (SH2-CrkII, Fig. 7D Coomassie) to lysates from EPEC-infected HeLa cells. Pull-downs were analyzed by blotting sequentially with anti-Tir and anti-GST MoAbs. We found that the presence of recombinant CrkII SH2 domain in the lysates blocked the binding of Nck SH2 domain to Tir in a concentration-dependent manner. These results provide further evidence that the SH2 domains of Crk and Nck compete for binding to Tir.

## Discussion

Numerous pathogens have evolved mechanisms to subvert host control of actin polymerization for their own benefit. EPEC manipulates the host actin cytoskeleton from outside the cell, making it a powerful model system to study eukaryotic phosphotyrosine signaling events in response to external stimuli [40]. Several host cell proteins implicated in cytoskeletal remodeling, including Crk adaptor proteins, are recruited to the site of EPEC attachment [12]. In the present study we aimed to investigate a possible role of Crk adaptors in pedestal formation by EPEC.

We inhibited CrkI/II and CrkL expression using siRNA in HeLa cells and then infected them with EPEC. We found that cells expressing reduced CrkI/II and CrkL formed increased numbers of pedestals than their control counterpart cells (Fig. 1). Next we used a complementary strategy (Fig. 2) in which we transfected a dominant-negative Crk mutant with an R38V mutation in the SH2 domain [36], previously shown to inhibit *Shigella* infection [41]. In parallel we overexpressed the WT CrkII isoform. We found that overexpression of WT or R38V protein did not alter the number of pedestals formed in HeLa cells. In addition, while the WT protein localized to pedestals, the R38V mutant did not.

To verify our results more rigorously, we measured the numbers of pedestals formed by EPEC infection of MEFs that were already deficient in CrkI/II or CrkL and in which expression of the remaining Crk isoform was suppressed using siRNA. The number of pedestals in these cells was significantly higher, than the number in control cells (Fig. 4, 5 and S3). This increase was due to more efficient pedestal formation (Fig. 4C). Pedestal number was also significantly higher in CrkI/II-deficient cells expressing the dominant-negative CrkII R38V mutant than in control cells (Fig. S3D, E). The results obtained with cells deficient in CrkI/II and CrkL was consistent with redundancy among Crk adaptors as suggested in other studies [22], [42]. Whereas these cell culture studies suggest similar ability of CrkI/II and CrkL to compensate for the absence of the other isoform, deletion of only CrkI/II [31] or CrkL [32] in mice is embryonically lethal. Thus, although CrkI/II and CrkL play overlapping roles, they may not be entirely functionally equivalent *in vivo*. Remarkably, we found that MEFs deficient in CrkI/II or CrkL upregulate the level of the remaining Crk isoform in a compensatory manner (Fig. S4). This is, to our knowledge, the first time Crk isoform expression has been measured in these cells, and the discovery was made possible with the help of a system which uses different colors to detect both isoforms simultaneously and unambiguously.

One of the major mechanisms regulating Crk adaptor proteins is phosphorylation of Tyr221 in CrkII and Tyr207 in CrkL in the linker region; this regulatory tyrosine is absent in CrkI. Phosphorylation of these tyrosines promotes their interaction with the SH2 domain in the same molecule, preventing the domain from binding other phosphotyrosines [25], [26]. These regulatory tyrosines are phosphorylated primarily by Abl kinase, which is activated upon EPEC infection [39]. For that reason we analyzed whether EPEC infection promotes phosphorylation of Crk adaptor proteins on these regulatory tyrosines. Indeed, we found that EPEC induced progressive phosphorylation of CrkII-Tyr221 and CrkL-Tyr207 that peaked at 2 h of infection in our experimental conditions (Fig. 6). Interestingly, the phospho-Crk proteins localized specifically to a thin interface between EPEC and pedestal rather than along the entire pedestal stalk (Figs. 6E). This is similar to the distribution of Abl kinase [39], [43] consistent with the idea that Abl phosphorylates Crk adaptors.

A remarkable finding in this study is that overexpression of the isolated SH2 domain of CrkII in HeLa cells let to a significant decrease in pedestal number (Fig. 3), just as overexpressing the SH2 domain of Nck, which binds to phosphorylated Tyr474 of Tir, inhibits pedestal formation by EPEC [6]. The SH2 domain of CrkII probably inhibits pedestal formation by interfering with Nck recruitment to Tir (Fig. 3), possibly because the SH2 domain of CrkII and CrkL adaptors interacts with Tir (Fig. 7A). This interaction is not observed with Tir carrying a Tyr474Phe mutation, suggesting that it occurs through the phosphorylated Tyr474 of Tir (Fig. 7B, C). In support of this idea, CrkII is not recruited to pedestals when the strain of EPEC expresses Tyr474Phe mutant Tir [12]. Since the SH2 domain of Grb2 adaptor does not bind Tir [37], not all SH2-containing proteins interact with phosphorylated Tir, suggesting that Crk binding to Tir reflects a specific regulatory event. Indeed, we competitively blocked the binding of Nck SH2 domain to Tir using recombinant CrkII SH2 domain. Taken together, these findings lead us to speculate that by binding to phosphorylated tyrosine 474 in Tir, Crk adaptors inhibit Nck recruitment and consequently actin polymerization.

At the same time, our findings show that Crk adaptors cannot substitute for Nck in promoting actin polymerization at pedestals. This is in agreement with the fact that Nck1/2-deficient cells expressing CrkII and CrkL do not form pedestals [6]. This highlights the many questions that remain regarding the factors regulating actin polymerization at pedestals. For example, it is not known whether recombinant Crk SH3 domain promotes N-WASP activation and Arp2/3 complex-mediated actin polymerization *in vitro,* similarly to other SH3 containing proteins. In addition, there is an Nck-independent pathway to actin polymerization that can be detected when the major Nck pathway is blocked [37]; indeed *in vivo* studies show that EPEC can use a variety of adhesion mechanisms that can compensate for the lack of Nck [44].

**Figure 8:**
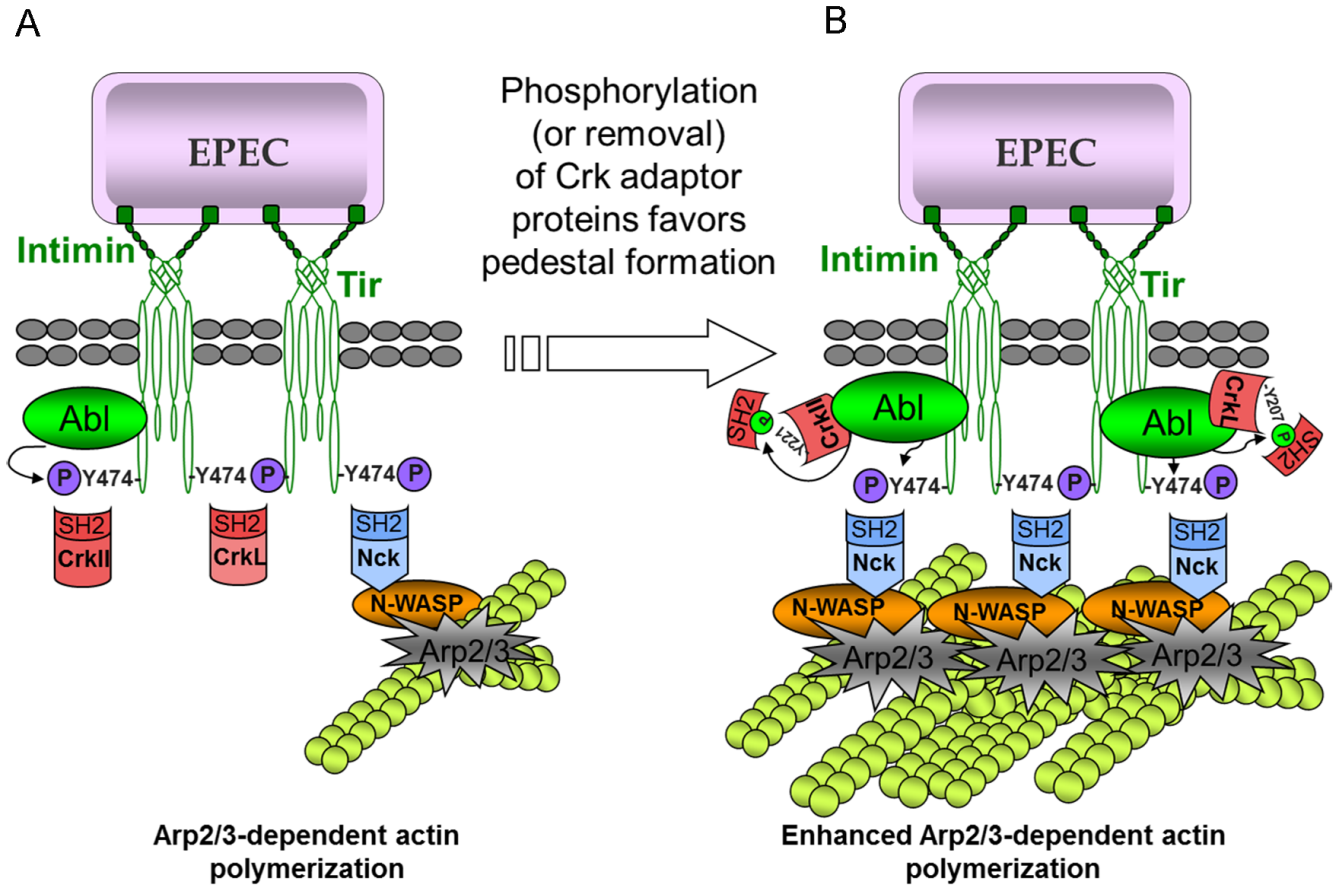
Model of the mechanism of action of Crk adaptor proteins in pedestal formation. (**A**) The functionally redundant Crk adaptor proteins inhibit pedestal formation, probably by competing with Nck for binding to Tir. Interestingly, Abl kinase, which phosphorylates CrkII, CrkL and Tir, has also been shown to associate with Tir through its SH3 domain. (**B**) Phosphorylation of Crk proteins by Abl, or the absence of Crk proteins, would leave more Tir molecules available for interacting with Nck, which would in turn promote actin polymerization by N-WASP and the Arp2/3 complex.

Based on the present study and previous work, we propose a model in which Crk and Nck adaptors compete for Tir binding in a stochastic fashion (Fig. 8). Tir clustering in the plasma membrane induces its phosphorylation on Tyr474, creating a docking site for the SH2 domain of Nck. Nck binding initiates the major Tir-Nck-N-WASP pathway to promote Arp2/3 complex-mediated actin polymerization [6], whereas binding by any one of the Crk isoforms inhibits Nck binding and therefore actin polymerization. This model can explain the significantly higher number of pedestals formed in the absence of all three Crk isoforms (Fig. 1, 4, 5, S3). It can also explain why during early infection, few Crk proteins are phosphorylated (Fig. 6) and so most should be available to compete with Nck for binding to Tir (Fig. 3 and 7). The model predicts that during later stages of infection, Abl can phosphorylate Crk adaptors and Tir, reducing the competition and promoting actin polymerization at pedestals. Consistent with this idea, preliminary studies show that anti-phospho-CrkL immunoprecipitates do not contain phospho-Tir (data not shown).

This model is consistent with the proposal that SH2-containing proteins bind transiently to phosphotyrosines in different target molecules (“hopping from binding site to binding site”) when these residues are present in high concentrations at the plasma membrane [45]. Such concentrations are likely to be present during EPEC infection after Tir has been phosphorylated. Our data are in agreement with this dynamic view of signaling involving proteins containing phosphotyrosines and SH2 domains.

## Material and Methods

### Cells, bacteria, reagents and antibodies

HeLa human epithelial cells were obtained from the American Type Culture Collection (ATCC). Wild-type (WT) and CrkI/II-deficient MEFs were obtained from Dr. Tom Curran (The Children's Hospital of Philadelphia, Pennsylvania, USA) [31], and WT and CrkL-deficient MEFs were obtained from Dr. Akira Imamoto (University of Chicago, Chicago, Illinois) [32]. Nck1/2-deficient MEFs were obtained from Dr. Anthony Pawson (Samuel Lunenfeld Research Institute Toronto, Canada). Cells were grown in Iscove's modified Dulbecco's medium (IMDM) supplemented with 10% fetal bovine serum (FBS) and penicillin/streptomycin. Enteropathogenic *Escherichia coli* (EPEC) E2348/69 and anti-Tir MoAb were from Dr. B. Brett Finlay (University of British Columbia, Vancouver, Canada). EPEC Δ*tir* mutant (strain E2348/69 with an in-frame deletion within the *tir* gene), which is resistant to nalidixic acid (25 μg/ml, final concentration) [5], and anti-Tir polyclonal Ab were provided by Dr. Brendan Kenny (University of Newcastle, Newcastle, UK).

The following commercial Abs were used: anti-Flag M2 mouse MoAb (Sigma), anti-GFP polyclonal and anti-GST monoclonal Abs (BD Biosciences and B14 MoAb from Santa Cruz Biotechnologies); anti-Myc tag 4A6 mouse MoAb (Merck); anti-CrkI/II mouse MoAb clone 22 (BD Biosciences Pharmingen); anti-CrkII rabbit polyclonal Ab (H53, Santa Cruz Biotechnology); and anti-CrkL rabbit polyclonal Ab (Ab C-20, Santa Cruz Biotechnology). For WB, phosphospecific rabbit MoAb against phosphorylated Tyr221 in CrkII (clone EPR269Y, Abcam) and phosphospecific polyclonal Ab against phosphorylated Tyr207 in CrkL (3181, Cell Signaling). For immunofluorescence studies, phosphospecific rabbit polyclonal Ab against phosphorylated Tyr221 in CrkII (3491, Cell Signaling) and phosphospecific polyclonal Ab against phosphorylated Tyr207 in CrkL (SAB4503814, Sigma) were used. Platinum anti-phosphotyrosine MoAb (Merck) was used to detect phosphorylated Tir.

Mouse MoAb against *E. coli* lipopolysaccharide (LPS) (clone 2D7/1) was from Abcam. Anti-β-actin C4 mouse MoAb was from MP Biomedicals. Anti-α-tubulin rat MoAb was from AbD Serotec. Anti-rabbit and anti-mouse horseradish peroxidase (HRP)-conjugated secondary Abs and ECL WB developer were from GE Healthcare Life Sciences. For WB experiments using the Odyssey infrared scanning system (Lycor, Fisher Scientific), Abs were purchased at a concentration of 1 mg/ml and used at 1:5,000 dilution. The following Abs were used for green signal: IRDye 800CW-labeled goat anti-rabbit and anti-mouse secondary Abs (Fisher Scientific). The following Abs were used for red signal: IRDye 680CW-labeled goat anti-rabbit Ab (Fisher Scientific) and Alexa 680-labeled goat anti-mouse Ab (Invitrogen, initial concentration 2 mg/ml, used at 1∶7,000). The following conjugated secondary abs were used for immunofluorescence studies: Alexa Fluor 488-labeled goat anti-mouse Ab and anti-rabbit Ab (green), Alexa Fluor 405-labeled goat anti-mouse Ab (blue) and 568-labeled goat anti-mouse Ab (red).

### Constructs

Myc-tagged rat WT CrkII, CrkII-R38V and CrkII-Y221F cDNAs cloned into the pCAGGS vector were obtained from Dr. Michiyuki Matsuda (Osaka University, Japan) [46]. The GST-tagged SH2 domain of Nck1 (residues 275-377) in pGEX-2T [47] and GST-tagged versions of CrkII (residues 1-124) and CrkL (residues 9-108) in pGEX-6P1 [27] were obtained from Dr. Bruce Mayer (University of Connecticut Health Center, Connecticut, USA). The plasmids pEBB-CrkII-SH2-GFP and pEBB-Flag-Nck2 were obtained from Dr. Gonzalo M. Rivera (Texas A&M University, Texas, USA). The plasmids p*tir* and p*tir*Y474F, which are pACYC184-based plasmids carrying the 3′ *map*, *tir* and *cesT* genes [48], were obtained from Dr. Brendan Kenny (University of Newcastle, Newcastle, UK). The plasmid p*tir*Y474F carries the Tyr474Phe substitution in Tir. Strains carrying pACYC184-based plasmids were generated by electroporation and selected with chloramphenicol (25 μg/ml, final concentration).

pSG5-CrkL (human) was obtained from Addgene (#29560, deposited by Dr Nora Heisterkamp, Children's Hospital, Los Angeles, USA). CrkL-Y207F was produced using the QuikChange site-directed mutagenesis kit (Stratagene), using the primer 5′GAACCTGCTCATGCATTCGCTCAACCTCAGACC3′. Mutants were verified by sequencing with the internal oligonucleotide 5′AAGGGTGAGATCCTAGTG3′.

### Preparation of electrocompetent EPEC cells and electroporation

Mid-log-phase cultures (50 mL, optical density at 600 nm  = 0.4–0.6) were harvested by centrifugation at 1,200 *g* for 15 min at 4°C, and the pellet was washed three times with 50 ml of cold sterile water. All manipulations were carried out on ice. The pellet was resuspended in 100 μl of cold sterile water. An aliquot (40 μl) of the resulting electrocompetent cells were electroporated immediately with 6 μl of DNA plasmid at 2 kV in electroporation cuvettes with a 1 mm gap (Cell Project) using a BTX Electro Cell Manipulator 600. Immediately following electroporation, cells were washed into the bottom of the electroporation chamber with 1 ml of SOC medium. The electroporated cells were allowed to recover for 1 h at 37°C, and then plated onto LB agar plates containing chloramphenicol.

### Cell transfection, siRNA treatment and EPEC pedestal formation

Cells were transfected with plasmids using Lipofectamine 2000 reagent or Lipofectamine LTX (Invitrogen). Briefly, HeLa cells were grown to 60–70% confluence in 6-well plates with or without heat-sterilized coverslips for immunofluorescence. Transfection was carried out for 20 h in IMDM containing 10% FBS without antibiotics.

Inhibition of human CrkI/II and CrkL expression by siRNA was carried out using sequence-specific oligonucleotides and a scrambled oligonucleotide as negative control. For experiments in Fig. S1 we used oligonucleotides from Santa Cruz Biotechnologies, while for experiments in Fig. 1 we used oligonucleotides from Ambion (Life Technologies, human anti-CrkI/II: s3520; human anti-CrkL: s3522) as per the manufacturer's instructions. Inhibition of mouse CrkL or CrkI/II expression using siRNA was carried out using two Silencer Pre-designed mouse siRNAs (s64409, n411975; s64406, s64404; Ambion) and a scrambled oligonucleotide (n411975). MEFs were grown to 50–60% confluence in 6-well plates and transfected with 40 nmoles of siRNA in the presence of 3 μl Lipofectamine RNAiMAX (Invitrogen) per well. Transfections were allowed to proceed for 20 h prior to EPEC infection. Changes in levels of Crk proteins were assessed by WB using anti-Crk Ab at 0.5 μg/ml or anti-CrkL Ab at 0.7 μg/ml.

WB experiments to control for siRNA treatment and transfection were performed using 6-well plates. Cells were washed once with cold Dulbecco's phosphate-buffered saline (D-PBS) with calcium and magnesium (Invitrogen) and scraped into 200–300 μl 2× Laemmli buffer. Samples were homogenized by 3 passages through a syringe with a 25-gauge needle and centrifugation at 21,000 *g* for 15 min at 4°C. Samples were resolved by 10% SDS-PAGE and analyzed by WB using the primary and secondary Abs described above. Densitometry of bands was carried out using NIH Image J software (Figs. S1). For other figures, membranes were incubated with the appropriate secondary Abs, then scanned with the Odyssey system using the red (700 nm) and green (800 nm) channels and quantitated with the Odyssey software.

For infections, EPEC was preactivated by incubating a 1∶100 dilution of an overnight culture for 2 h in IMDM supplemented with 10% FBS without antibiotics at 37°C and 5% CO_2_. After the preactivation, the optical density of the suspension at 600 nm was adjusted to 0.2. Cells were infected at the indicated multiplicity of infection (MOI) and allowed to form pedestals for the indicated time in IMDM supplemented with 10% FBS without antibiotics. MOIs were adjusted to take into account that EPEC pedestal formation is less efficient on murine cells than on human cells; and for practical reasons, considering the protocol to be performed. Therefore MOIs were much lower in experiments to quantify pedestal number than in pull-down assays (see below). Pedestals were counted in representative fields containing a total of 100 cells. Experiments were performed at least three times.

### Detection of phosphorylated Crk proteins

HeLa cells were trypsinized, seeded at 80–90% confluence in 150 mm plates and allowed to attach for 8 h. Cells were washed once with serum-free medium and starved in this medium for 16 h until infection. Preactivated EPEC was washed once by centrifugation at 1,800 *g* for 5 min to remove serum. Monolayers were then infected in the absence of serum with EPEC at an MOI of 45 for 1, 2 or 3 h or left uninfected as a control. Cells were washed once in cold D-PBS and lysed in modified RIPA buffer [50 mM Tris-HCl (pH 7.4), 150 mM NaCl, 15% glycerol, 2 mM EDTA, 0.1% SDS, 1% Triton X-100, 1 mM Na_3_VO_4_, 10 mM NaF, 1 mM PMSF, protease inhibitor cocktail (Amersham), and phosphatase inhibitor (PhosSTOP, Roche)]. Samples were homogenized by 3 passages through a syringe with a 25-gauge needle and centrifuged at centrifuged at 21,000 *g* for 10 min at 4°C. For CrkII immunoprecipitations, 2.5 μg of anti-CrkII MoAb or IgG isotype control were incubated 1 h with 30 μl of magnetic beads coated with anti-mouse IgG. After one wash, the beads were added to the lysates and incubated with tumbling for 4 h at 4°C. For WB experiments, the clarified lysates were mixed with Laemmli sample buffer, boiled for 5 min, subjected to 10% SDS-PAGE and analyzed by WB using the primary and secondary Abs described above.

### Immunofluorescence microscopy

Cells were fixed with 10% formalin solution (formaldehyde 4% w/v, Sigma) in PBS at room temperature and permeabilized with 0.1% Triton X-100 for 5 min. After three washes with PBS, cells were blocked with 2% BSA in PBS for 10 min and then sequentially stained with 1 μg/ml tetramethyl rhodamine isothiocyanate (TRITC)-phalloidin (Sigma) or Alexa 350-labeled phalloidin (Invitrogen) to visualize filamentous actin (F-actin). Bacteria were visualized with DAPI (300 nM) or with MoAb against *E. coli* LPS (clone 2D7/1, Abcam) at a final concentration of 5 μg/ml, followed by Alexa 405-conjugated goat anti-mouse Ab (diluted 1∶750) for detection in the blue channel.

Photographs were taken on a Nikon Eclipse TE 200-U fluorescence microscope using a Hamamatsu camera. Images were processed with Adobe Photoshop (Fig. S2). Confocal microscopy in Figs.1, 4, 5 and 6 was performed at the Parque Científico de Madrid microscopy facility using a Leica Confocal SP2/DM-IRE2 and Leica software (version 2.61). Images in Figs. 2, 3, S3, and S5 were acquired on a Zeiss AX10 Imager A.1 fluorescence microscope equipped with an AxioCam MRm camera and AxioVision Release 4.7 software. Experiments were performed at least three times.

### Pull-down experiments

GST and the GST-SH2 domains of Nck, CrkII and CrkL were produced in *E. coli* BL21, then purified and coupled to GSH beads (GE Healthcare Life Sciences) by standard protocols as previously described [49]. HeLa cells were grown on 150-mm plates to 70–80% confluence and infected at an MOI of 180 for 1 or 2 h, washed three times with D-PBS and scraped into 600 μl of modified RIPA buffer. The GST-fusion proteins were added to 150 μl of cell lysate from each condition and incubated for 5 h with tumbling at 4°C. Pull-downs were washed four times with 200 μl lysis buffer diluted 1∶10 in PBS as described [50].

For competitive pull-downs, the isolated SH2 domain of CrkII was excised from the GST-SH2 CrkII fusion protein coupled to GSH beads using the PreScission protease according to the manufacturer's instructions (GE Healthcare Life Sciences). Protein concentration was determined using the DC protein Assay kit (Biorad) and adjusted to 0.5 mg/ml using PBS. The indicated amounts of soluble SH2 domain were added to 100 μl lysates from infected HeLa cells at an MOI of 15. After adjusting the final volume to 200 μl with PBS, the lysates were incubated for 2 hours at 4°C. Then, pull-downs were performed using GSH beads carrying the GST-tagged Nck2 SH2 domain (5 μg) or GST alone (12 μg) for 4 h at 4°C with tumbling. Pull-downs were washed twice with 200 μl lysis buffer diluted 1∶10 in PBS, reconstituted in 50 μl of 2× Laemmli sample buffer and 30 μl were loaded in a gel for WB analysis.

### Statistical methods

Statistical analysis was performed using GraphPad Prism software (version 5.0).

### Accession numbers for genes/proteins used in this study

**Tir (EPEC).** Complete genome, strain E2348/69: GenBank: FM180568.1

“Translocated Intimin Receptor” NCBI Gene ID: AF013122.

**Nck1/2.** *Nck1:* Nck1 non-catalytic region of tyrosine kinase adaptor protein 1 (Mus musculus); NCBI Gene ID: 17973.

*Nck2:* Nck2 non-catalytic region of tyrosine kinase adaptor protein 2 (Mus musculus); NCBI Gene ID: 17974.

*Nck2*: NCK adaptor protein 2 (Homo sapiens) NCBI Gene ID: 8440.

**CrkI/II and CrkL.** *Crk*I: adapter molecule crk isoform 1 (Mus musculus); NCBI Gene ID: 12928.

*CrkII*: adapter molecule crk isoform 2 (Mus musculus); NCBI Gene ID: 12928.

*CrkII*: Crk v-crk avian sarcoma virus CT10 oncogene homolog (Rattus norvegicus); NCBI Gene ID: 54245

*CrkL*: Crkl v-crk sarcoma virus CT10 oncogene homolog (avian)-like (Mus musculus); NCBI Gene ID: 12929.

*CrkL* v-crk avian sarcoma virus CT10 oncogene homolog-like (Homo sapiens); NCBI Gene ID: 1399. Addgene: https://www.addgene.org/29560/

## Supporting Information

Figure S1 **Pedestal formation in HeLa cells in which either CrkI/II or CrkL expression is inhibited by siRNA.** WB of HeLa cell extracts using **(A)** anti-CrkI/II Ab and chemiluminescence or **(B)** anti-CrkL Ab and the Odyssey imaging system, showing siRNA-induced expression inhibition of the indicated proteins in infected cells. As a loading control, the blots were probed with anti-actin MoAb or anti-tubulin Ab. **(C, D)** Quantitation of the number of pedestals on infected HeLa cells pretreated by siRNA against CrkI/II, CrkL or a control oligonucleotide. Quantitation was done by counting the number of pedestals on 100 cells. Data in the graphs show mean ± standard deviation (SD) for three independent experiments. The difference between groups was not statistically significant based on Student's *t*-test.
(TIF)

Figure S2 **Pedestal formation in MEFs deficient in CrkI/II or CrkL. (A)** WT and CrkI/II-deficient MEFs were analyzed by WB using anti-CrkII Ab to show endogenous expression levels and reconstitution with transfected Myc-tagged WT CrkII (R CrkII). As a loading control, the blot was probed with anti-actin MoAb (upper bands). **(B)** Fluorescence images of MEFs infected with preactivated EPEC for 3 h at an MOI of 3. Actin was stained red using TRITC-phalloidin, and bacteria were stained blue using DAPI. The merged images for all conditions are also shown. Transfected cells were stained with anti-Myc MoAb, followed by Alexa 488-conjugated goat anti-mouse secondary Ab. The merged images shown were generated using Adobe Photoshop. **(C)** Quantitation of the number of pedestals formed on WT MEFs, CrkI/II-deficient MEFs and rescued CrkII cells (R CrkII). **(D)** WT and CrkL-deficient MEFs were analyzed by WB using anti-CrkL Ab and anti-CrkII MoAb to show endogenous expression levels. **(E)** Quantitation of the number of pedestals formed on WT, CrkL-deficient and CrkL rescued MEFs (R CrkL). Quantitations were done by counting the number of pedestals on 100 cells. The graphs show mean ± standard deviation (SD) for three independent experiments. The differences among groups were not statistically significant based on Student's *t*-test.
(TIF)

Figure S3 **Absence or inhibition of all Crk isoforms potentiates pedestal formation. (A)** WT and CrkI/II-deficient MEFs were analyzed by WB using anti-CrkL Ab to show that CrkL levels were lower in cells treated with a siRNA against CrkL than in cells treated with a scrambled control oligonucleotide (lower bands). As a loading control, the blots were probed with anti-tubulin Ab (upper bands). **(B)** Fluorescence images of WT and CrkI/II-deficient MEFs in which CrkL expression was inhibited by siRNA; cells were infected with preactivated EPEC for 3 h at an MOI of 3. Actin was stained red using TRITC-phalloidin, while EPEC was stained blue using DAPI. Images were merged using Axio Vision software. Insets are 4× digital zoom images. **(C)** Quantitation of the number of pedestals on WT and CrkI/II-deficient cells treated for siRNA using a scrambled control (Ctr.) oligonucleotide (white bar) or an oligonucleotide to reduce CrkL expression (back bar). Quantitation was done by counting the number of pedestals on 100 cells. The graph shows mean ± standard deviation (SD) for three independent experiments. The indicated groups differed significantly based on Student's *t*-test. **, p<0.01. **(D)** Pedestal formation in CrkI/II-deficient cells expressing a dominant-negative CrkII mutant. Expression in transfectants was assessed by WB with anti-Myc MoAb. The blot was also probed with anti-CrkII MoAb to show endogenous expression of CrkII and expression of dominant-negative R38V Crk mutant (arrows). As a loading control the blot was probed with anti-actin MoAb (upper bands). The merge of both images is also shown. **(E)** Quantitation of the number of pedestals on mock-treated WT cells (white bar), mock-treated CrkI/II-deficient cells (black bar), and CrkI/II-deficient cells transfected with the dominant-negative mutant (R38V, grey bar); all cells were infected with preactivated EPEC for 3 h at an MOI of 3. Quantitation was done by counting the number of pedestals on 100 cells. The graph shows mean ± SD for three independent experiments. Statistical analysis using Student's *t*-test is shown. *, p<0.05.
(TIF)

Figure S4 **Analysis of CrkL expression in CrkI/II-deficient fibroblasts and of CrkII expression in CrkL-deficient fibroblasts. (A)** WB of lysates from WT and CrkI/II-deficient MEFs using anti-CrkII and anti-CrkL Abs to show expression levels of CrkII and CrkL proteins. The merge of both images is also shown. **(B)** The graph shows mean ± SD values for the ratio of CrkII to actin signals for four independent experiments. Statistical analysis was carried out using Student's *t*-test. a.u.: arbitrary units, *, p<0.05. **(C)** WB of lysates from WT and CrkL-deficient MEFs using anti-CrkII and anti-CrkL Abs to show CrkII and CrkL levels. The merge of both images is also shown. **(D)** Graph shows mean ± SD values for the ratio of CrkL to actin signals for three independent experiments. Statistical analysis was carried out using Student's *t*-test. a.u.: arbitrary units, **, p<0.01.
(TIF)

Figure S5 **Immunofluorescence staining of Nck1/2-deficient MEFs cotransfected with Nck2 and CrkII SH2 domain.** Transfected MEFs were infected with EPEC at an MOI of 225. Cells were stained with anti-Flag MoAb followed by Alexa 568-conjugated goat anti-mouse Ab. EPEC was visualized with DAPI. GFP expression is shown in green. Images were merged using Axio Vision software. Insets are 4× digital zoom images.
(TIF)
