# Supplementary figures and images for: Crk Adaptors Negatively Regulate Actin Polymerization in Pedestals Formed by Enteropathogenic Escherichia coli (EPEC) by Binding to Tir Effector
Source: PLoS Pathog. 2014 Mar 27;10(3):e1004022. doi: 10.1371/journal.ppat.1004022 (PMC3968158; doi:10.1371/journal.ppat.1004022)

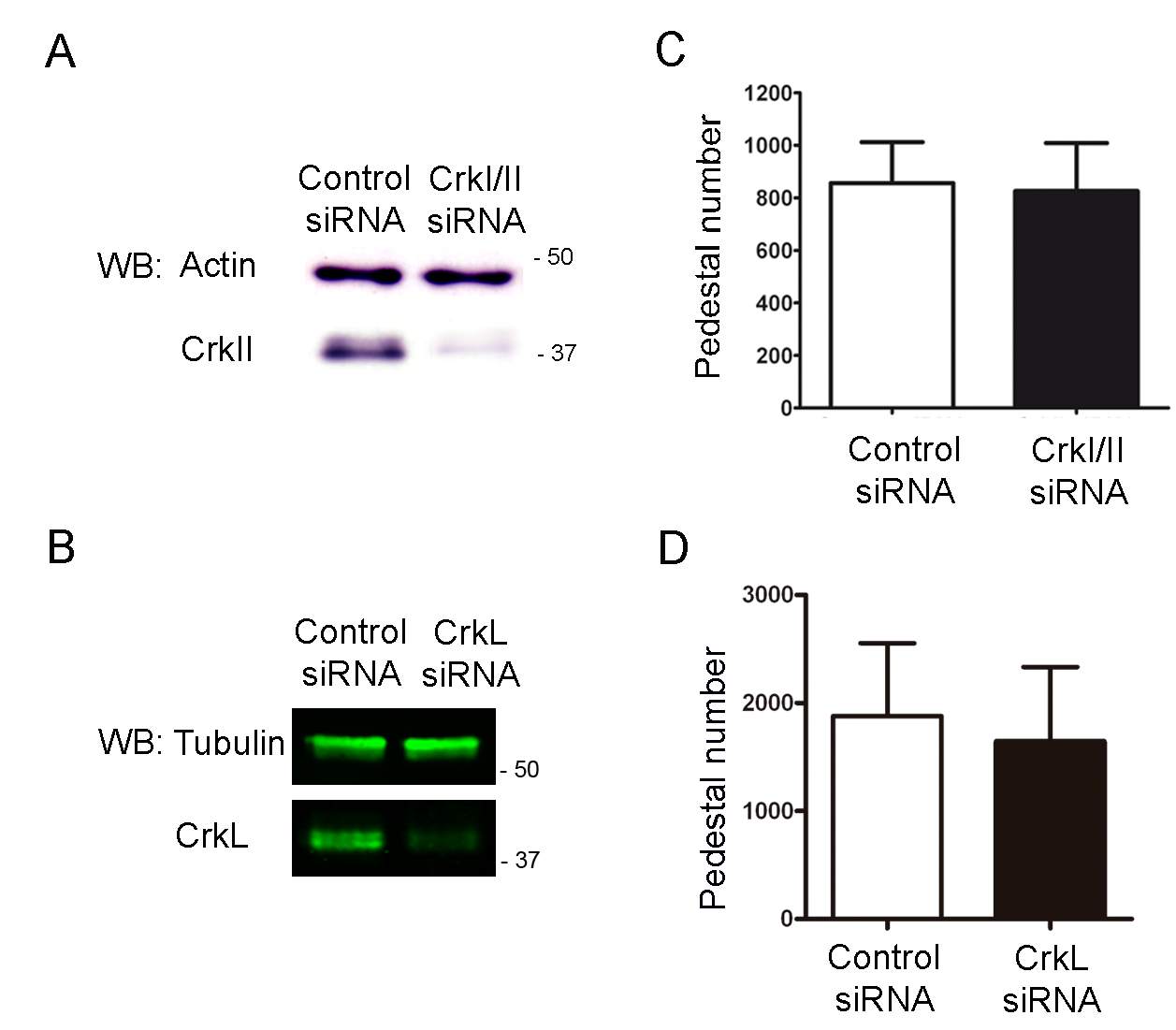

Supplement: Figure S1 — Pedestal formation in HeLa cells in which either CrkI/II or CrkL expression is inhibited by siRNA. WB of HeLa cell extracts using (A) anti-CrkI/II Ab and chemiluminescence or (B) anti-CrkL Ab and the Odyssey imaging system, showing siRNA-induced expression inhibition of the indicated proteins in infected cells. As a loading control, the blots were probed with anti-actin MoAb or anti-tubulin Ab. (C, D) Quantitation of the number of pedestals on infected HeLa cells pretreated by siRNA against CrkI/II, CrkL or a control oligonucleotide. Quantitation was done by counting the number of pedestals on 100 cells. Data in the graphs show mean ± standard deviation (SD) for three independent experiments. The difference between groups was not statistically significant based on Student's t-test. (TIF) [file ppat.1004022.s001.tif]

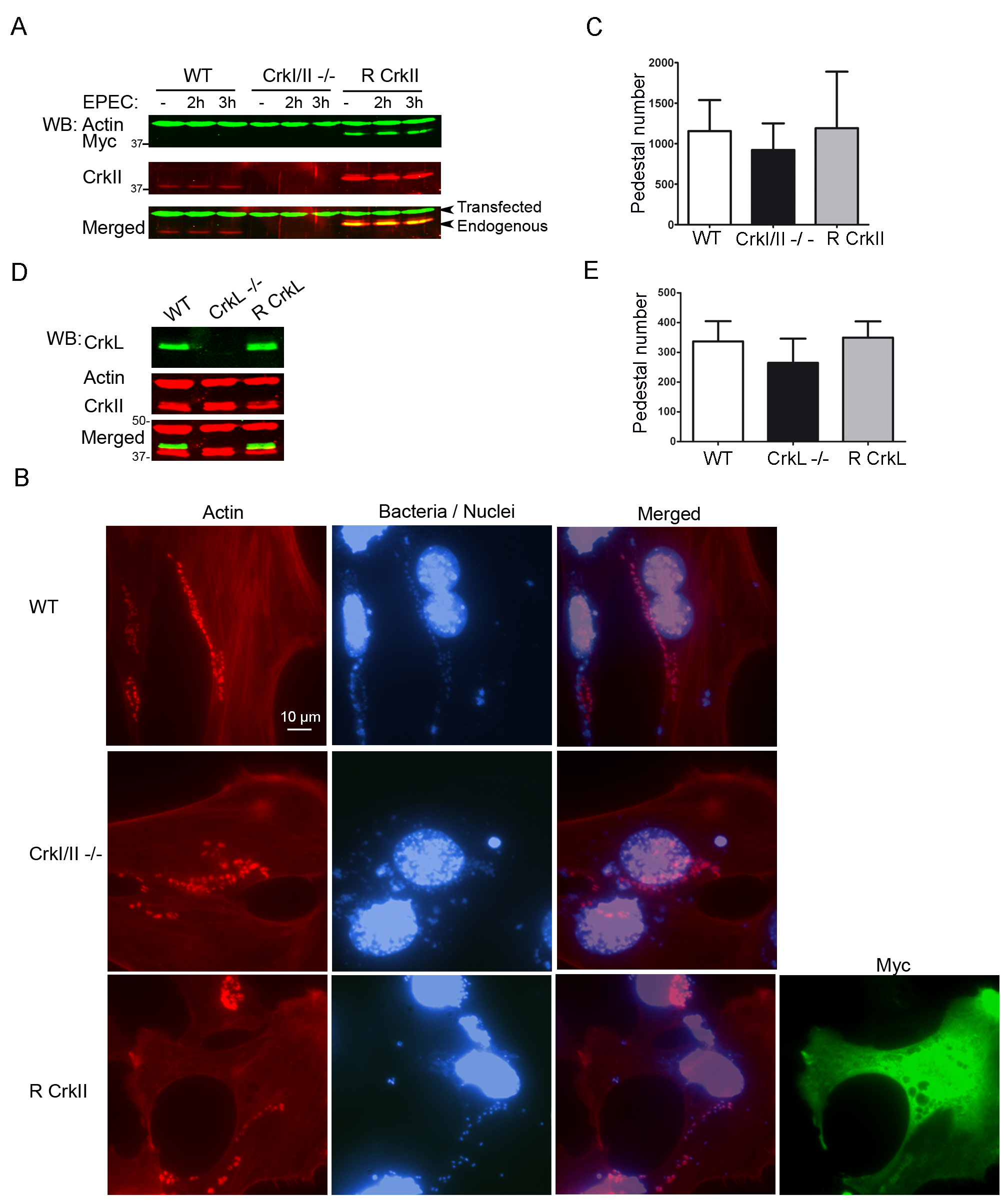

Supplement: Figure S2 — Pedestal formation in MEFs deficient in CrkI/II or CrkL. (A) WT and CrkI/II-deficient MEFs were analyzed by WB using anti-CrkII Ab to show endogenous expression levels and reconstitution with transfected Myc-tagged WT CrkII (R CrkII). As a loading control, the blot was probed with anti-actin MoAb (upper bands). (B) Fluorescence images of MEFs infected with preactivated EPEC for 3 h at an MOI of 3. Actin was stained red using TRITC-phalloidin, and bacteria were stained blue using DAPI. The merged images for all conditions are also shown. Transfected cells were stained with anti-Myc MoAb, followed by Alexa 488-conjugated goat anti-mouse secondary Ab. The merged images shown were generated using Adobe Photoshop. (C) Quantitation of the number of pedestals formed on WT MEFs, CrkI/II-deficient MEFs and rescued CrkII cells (R CrkII). (D) WT and CrkL-deficient MEFs were analyzed by WB using anti-CrkL Ab and anti-CrkII MoAb to show endogenous expression levels. (E) Quantitation of the number of pedestals formed on WT, CrkL-deficient and CrkL rescued MEFs (R CrkL). Quantitations were done by counting the number of pedestals on 100 cells. The graphs show mean ± standard deviation (SD) for three independent experiments. The differences among groups were not statistically significant based on Student's t-test. (TIF) [file ppat.1004022.s002.tif]

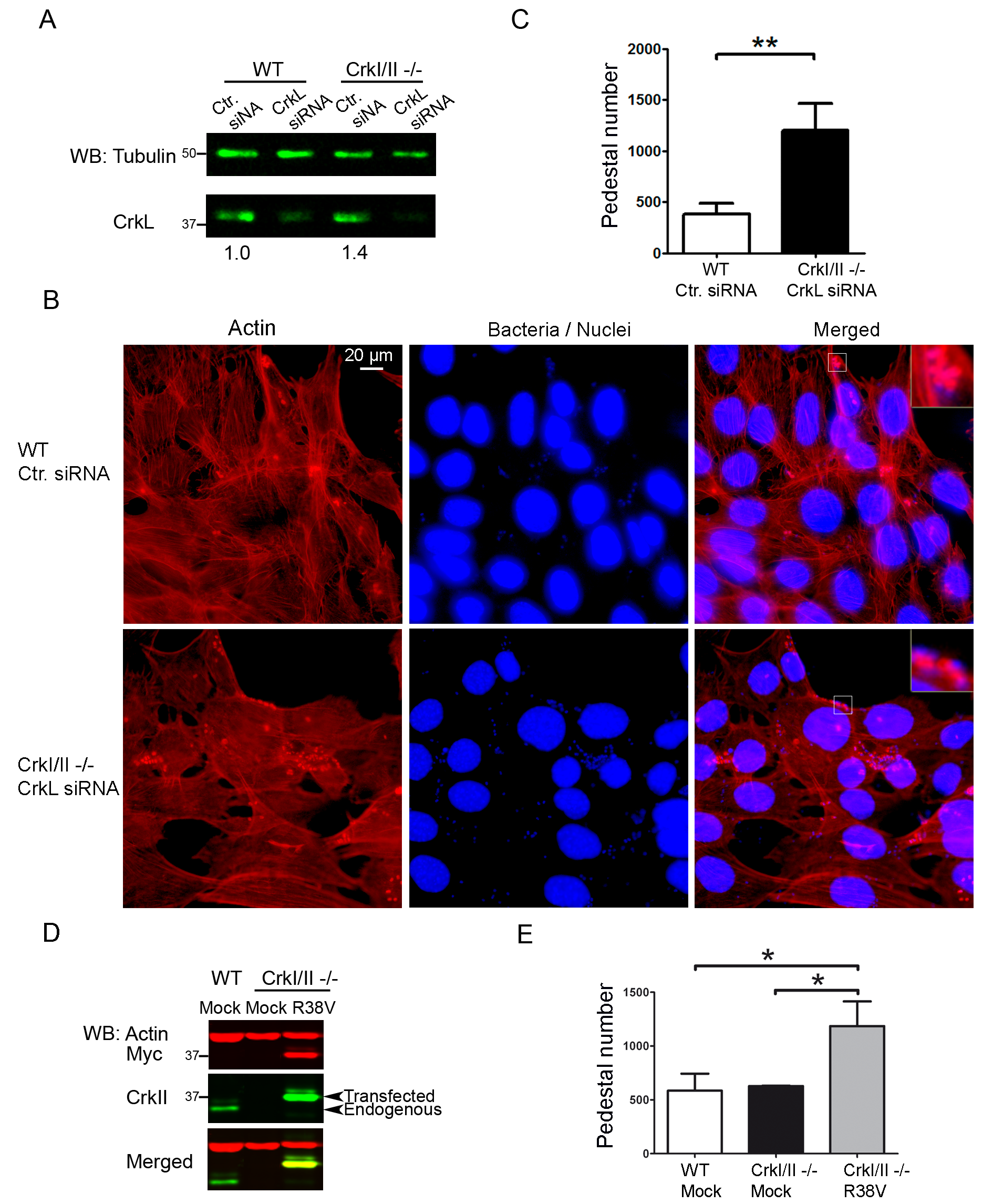

Supplement: Figure S3 — Absence or inhibition of all Crk isoforms potentiates pedestal formation. (A) WT and CrkI/II-deficient MEFs were analyzed by WB using anti-CrkL Ab to show that CrkL levels were lower in cells treated with a siRNA against CrkL than in cells treated with a scrambled control oligonucleotide (lower bands). As a loading control, the blots were probed with anti-tubulin Ab (upper bands). (B) Fluorescence images of WT and CrkI/II-deficient MEFs in which CrkL expression was inhibited by siRNA; cells were infected with preactivated EPEC for 3 h at an MOI of 3. Actin was stained red using TRITC-phalloidin, while EPEC was stained blue using DAPI. Images were merged using Axio Vision software. Insets are 4× digital zoom images. (C) Quantitation of the number of pedestals on WT and CrkI/II-deficient cells treated for siRNA using a scrambled control (Ctr.) oligonucleotide (white bar) or an oligonucleotide to reduce CrkL expression (back bar). Quantitation was done by counting the number of pedestals on 100 cells. The graph shows mean ± standard deviation (SD) for three independent experiments. The indicated groups differed significantly based on Student's t-test. **, p<0.01. (D) Pedestal formation in CrkI/II-deficient cells expressing a dominant-negative CrkII mutant. Expression in transfectants was assessed by WB with anti-Myc MoAb. The blot was also probed with anti-CrkII MoAb to show endogenous expression of CrkII and expression of dominant-negative R38V Crk mutant (arrows). As a loading control the blot was probed with anti-actin MoAb (upper bands). The merge of both images is also shown. (E) Quantitation of the number of pedestals on mock-treated WT cells (white bar), mock-treated CrkI/II-deficient cells (black bar), and CrkI/II-deficient cells transfected with the dominant-negative mutant (R38V, grey bar); all cells were infected with preactivated EPEC for 3 h at an MOI of 3. Quantitation was done by counting the number of pedestals on 100 cells. The graph shows mean ± SD fo [file ppat.1004022.s003.tif]

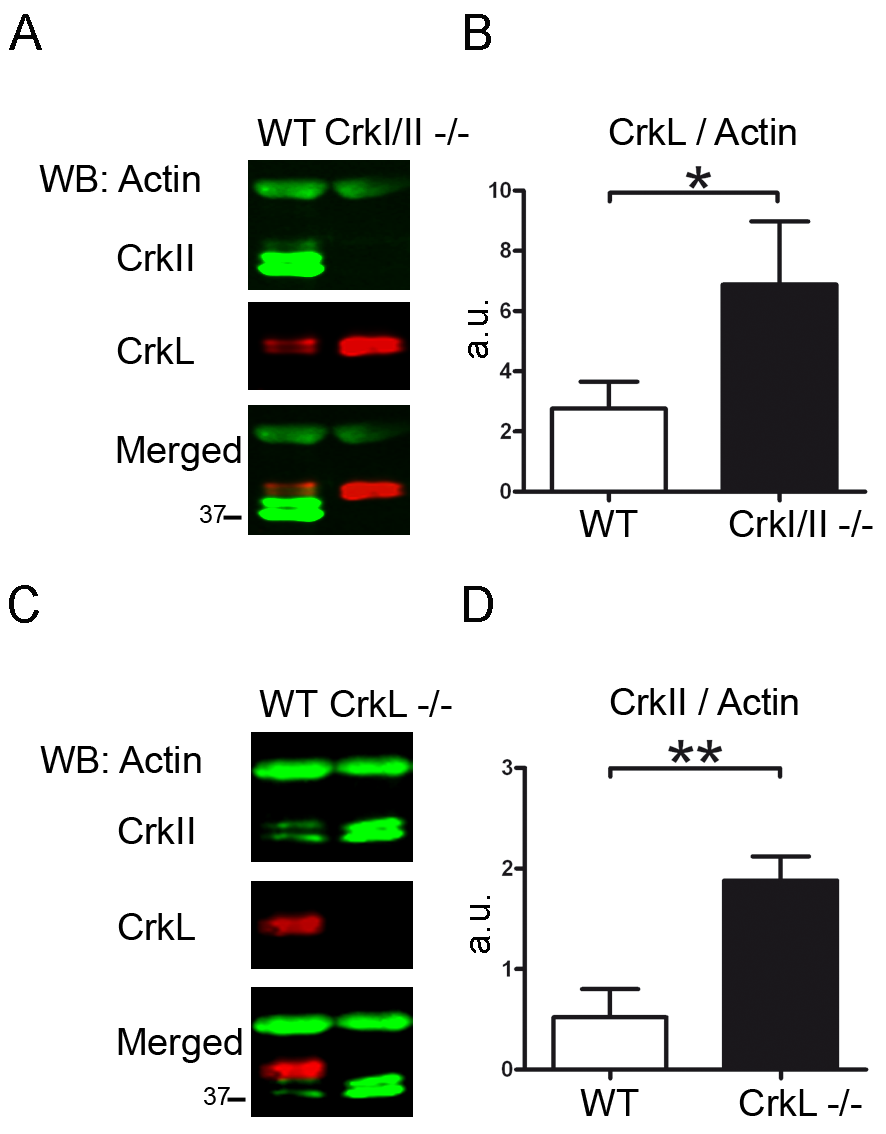

Supplement: Figure S4 — Analysis of CrkL expression in CrkI/II-deficient fibroblasts and of CrkII expression in CrkL-deficient fibroblasts. (A) WB of lysates from WT and CrkI/II-deficient MEFs using anti-CrkII and anti-CrkL Abs to show expression levels of CrkII and CrkL proteins. The merge of both images is also shown. (B) The graph shows mean ± SD values for the ratio of CrkII to actin signals for four independent experiments. Statistical analysis was carried out using Student's t-test. a.u.: arbitrary units, *, p<0.05. (C) WB of lysates from WT and CrkL-deficient MEFs using anti-CrkII and anti-CrkL Abs to show CrkII and CrkL levels. The merge of both images is also shown. (D) Graph shows mean ± SD values for the ratio of CrkL to actin signals for three independent experiments. Statistical analysis was carried out using Student's t-test. a.u.: arbitrary units, **, p<0.01. (TIF) [file ppat.1004022.s004.tif]

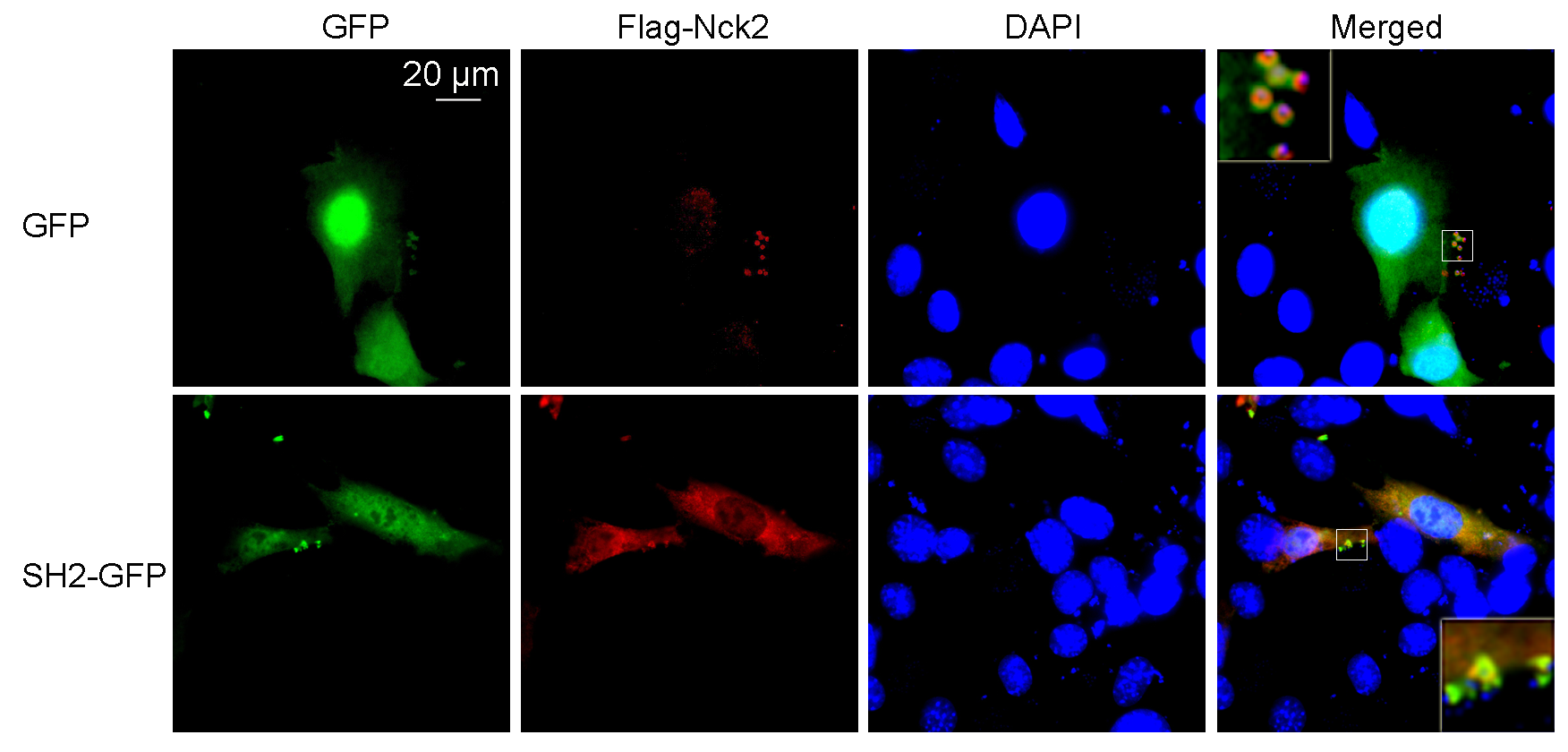

Supplement: Figure S5 — Immunofluorescence staining of Nck1/2-deficient MEFs cotransfected with Nck2 and CrkII SH2 domain. Transfected MEFs were infected with EPEC at an MOI of 225. Cells were stained with anti-Flag MoAb followed by Alexa 568-conjugated goat anti-mouse Ab. EPEC was visualized with DAPI. GFP expression is shown in green. Images were merged using Axio Vision software. Insets are 4× digital zoom images. (TIF) [file ppat.1004022.s005.tif]
